# Three-dimensional structured illumination microscopy with enhanced axial resolution

**DOI:** 10.1038/s41587-022-01651-1

**Published:** 2023-01-26

**Authors:** Xuesong Li, Yicong Wu, Yijun Su, Ivan Rey-Suarez, Claudia Matthaeus, Taylor B. Updegrove, Zhuang Wei, Lixia Zhang, Hideki Sasaki, Yue Li, Min Guo, John P. Giannini, Harshad D. Vishwasrao, Jiji Chen, Shih-Jong J. Lee, Lin Shao, Huafeng Liu, Kumaran S. Ramamurthi, Justin W. Taraska, Arpita Upadhyaya, Patrick La Riviere, Hari Shroff

**Affiliations:** 1 Laboratory of High Resolution Optical Imaging, National Institute of Biomedical Imaging and Bioengineering, National Institutes of Health, Bethesda, MD USA; 2 Present Address: Advanced Imaging and Microscopy Resource, National Institutes of Health, Bethesda, MD USA; 3 Leica Microsystems, Inc., Deerfield, IL USA; 4 SVision, LLC, Bellevue, WA USA; 5 Institute for Physical Science and Technology, University of Maryland, College Park, MD USA; 6 Biochemistry and Biophysics Center, National Heart, Lung, and Blood Institute, National Institutes of Health, Bethesda, MD USA; 7 Laboratory of Molecular Biology, National Cancer Institute, National Institutes of Health, Bethesda, MD USA; 8 Section on Biophotonics, National Institute of Biomedical Imaging and Bioengineering, National Institutes of Health, Bethesda, MD USA; 9 State Key Laboratory of Modern Optical Instrumentation, College of Optical Science and Engineering, Zhejiang University, Hangzhou, China; 10 Department of Neuroscience and Department of Cell Biology, Yale University School of Medicine, New Haven, CT USA; 11 Department of Physics, University of Maryland, College Park, MD USA; 12 Department of Radiology, University of Chicago, Chicago, IL USA; 13 MBL Fellows, Marine Biological Laboratory, Woods Hole, MA USA; 14 Present Address: Janelia Research Campus, Howard Hughes Medical Institute (HHMI), Ashburn, VA USA; 15 Present Address: State Key Laboratory of Modern Optical Instrumentation, College of Optical Science and Engineering, Zhejiang University, Hangzhou, China

**Keywords:** Super-resolution microscopy, Biotechnology, Super-resolution microscopy

## Abstract

The axial resolution of three-dimensional structured illumination microscopy (3D SIM) is limited to ∼300 nm. Here we present two distinct, complementary methods to improve axial resolution in 3D SIM with minimal or no modification to the optical system. We show that placing a mirror directly opposite the sample enables four-beam interference with higher spatial frequency content than 3D SIM illumination, offering near-isotropic imaging with ∼120-nm lateral and 160-nm axial resolution. We also developed a deep learning method achieving ∼120-nm isotropic resolution. This method can be combined with denoising to facilitate volumetric imaging spanning dozens of timepoints. We demonstrate the potential of these advances by imaging a variety of cellular samples, delineating the nanoscale distribution of vimentin and microtubule filaments, observing the relative positions of caveolar coat proteins and lysosomal markers and visualizing cytoskeletal dynamics within T cells in the early stages of immune synapse formation.

## Abstract (web summary)

Enhanced axial resolution for 3D SIM is achieved with deep learning or four-beam interference.

## Main

Three-dimensional structured illumination microscopy (3D SIM^1^) excites the sample with non-uniform illumination, providing information outside the diffraction-limited passband that is encoded in the fluorescence captured by diffraction-limited images. Decoding this extra information mathematically yields a super-resolution reconstruction with doubled resolution compared to wide-field microscopy. Although a more modest gain than other methods^2^, in thin samples 3D SIM offers advantages including good optical sectioning, low illumination dose and high acquisition speed (enabling ‘4D’ volumetric time-lapse imaging in living cells^3,4^) and compatibility with arbitrary fluorophores (facilitating multi-color super-resolution imaging^5^). These attributes have provided a plethora of biological insights^6–19^.

Although superior to wide-field microscopy, 3D SIM’s axial resolution is still limited to ∼300 nm, considerably worse than its ∼120-nm lateral resolution. Thus, 3D SIM reconstructions are anisotropic, distorting and obscuring fine features along the axial dimension. Relatively few solutions exist^20,21^ for reducing this anisotropy, and none has been widely adopted.

Here we demonstrate two complementary methods for improving axial resolution, with minimal or no modification to the 3D SIM optical path. First, we show that placing a mirror directly opposite the sample enables four-beam interference, producing higher axial spatial frequency components in the illumination pattern. This enables an axial resolution of ∼160 nm, producing nearly isotropic reconstructions. Second, we developed improved deep learning algorithms that operate on 3D SIM data (without the mirror), producing reconstructions with isotropic ∼120-nm spatial resolution. This computational method may be further combined with denoising, enabling high-quality, isotropic, 4D super-resolution imaging over dozens of volumes. We demonstrate these methods on a variety of fixed and live cellular samples. In vegetative and sporulating bacteria, we visualized membranes, cell division proteins and core components of the spore coat. In eukaryotic cells, we inspected membrane-encased actin filaments and pores that traversed thin membrane extensions; delineated the nanoscale positioning of vimentin and microtubules; observed the spatial distribution of caveolar coat proteins; and performed time-lapse volumetric imaging of mitochondrial, lysosomal and cytoskeletal dynamics.

## Results

### Four-beam interference for higher axial resolution

**Extended Data Fig. 1:**
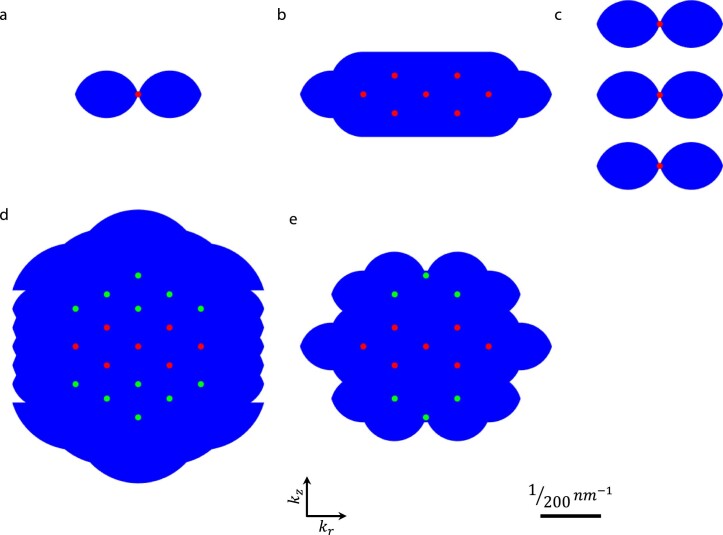
Support of optical transfer functions assuming a silicone oil objective lens. Axial cross sections through support are shown for **a**) wide-field, **b**) 3D SIM, **c)** standing wave, **d**) I^5^S, and **e**) four-beam, mirror-based SIM systems. Dots indicate illumination spatial frequency components, with green dots in **d, e**) indicating additional frequency components that are not present in 3D SIM. Supports were simulated with the following parameters: emission NA = 1.35; refractive index of immersion medium = 1.406; excitation wavelength: 488 nm; emission wavelength: 525 nm; side beams in **b, d, e**) at 92% of pupil radius.

Spatial resolution and optical sectioning in 3D SIM are determined by the convolution of the structured illumination pattern’s spatial frequency components with the wide-field detection optical transfer function (OTF; Extended Data Fig. 1a,b)^1^. Anisotropic spatial resolution is a consequence of limited angular aperture: (1) the three wave vectors whose interference produces the illumination pattern lie on a spherical cap with limited angular extent; and (2) the limited angular range over which fluorescence is collected produces an OTF with greater lateral than axial extent. We, thus, considered strategies to increase angular aperture to improve axial resolution in 3D SIM.

If the angle between illumination beams is increased beyond the limit imposed by the numerical aperture (NA) of the objective lens^22,23^, the resulting interference may contain higher axial frequency components up to 2n/λ, where n is the refractive index (RI) and λ is the wavelength of illumination. Such ‘standing-wave microscopes’^23^—for very thin samples—indeed unveil axial detail obscured in 3D SIM. For thicker samples, however, gaps in the OTF support (Extended Data Fig. 1c) preclude optical sectioning and generate severe axial ‘ringing’ in the resulting images.

These problems are resolved in I^5^S (ref. ^20^), wherein two opposed objectives introduce six coherent illumination wave vectors, yielding more frequency components than in 3D SIM. The same objectives are used to collect fluorescence, which is also coherently interfered. The combination of coherent illumination and detection (Extended Data Fig. 1d) produces ∼100-nm isotropic spatial resolution. However, I^5^S also presents severe practical challenges. (1) The illumination and fluorescence paths require more optics than 3D SIM, adding complexity and diminishing sensitivity. (2) Due to the short fluorescence coherence length, emission paths must be aligned to near-zero path length difference and maintained to much better than λ. In practice, this requires active feedback, further adding to instrument complexity. (3) Small RI mismatches introduce substantial aberrations. These reasons might explain why the only demonstration of I^5^S on biological samples^20^ was limited to small imaging fields, fixed cells with special mounting protocols and single-color applications.

An intriguing alternative to I^5^S was recently proposed^21^, whereby the 3D SIM central illumination beam is isolated, re-imaged to a mirror and reflected back toward the sample, yielding a four-beam interference pattern with finer axial structure than in 3D SIM (Extended Data Fig. 1e). If a high NA objective lens is used to collect the fluorescence, the theoretically predicted axial resolution is worse than I^5^S but substantially better than 3D SIM.

Although much simpler than I^5^S, notable challenges still exist. First, additional optics are still required, adding considerable complexity relative to the 3D SIM optical path. Second, the reflected beam must traverse these optics, air, sample and buffer, introducing RI mismatches that add undesirable wavefront distortion. Third, the additional optical path length would span almost a meter, implying that the illumination source must have a coherence length of at least this length, so that interference between direct and reflected beams is possible. This requirement rules out common laser sources used in microscopy.

**Fig. 1:**
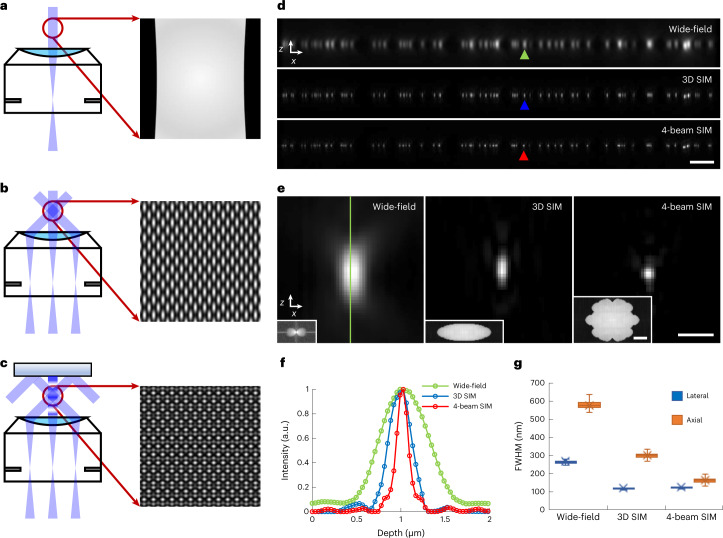
Improving axial resolution in 3D SIM. **a**–**c**, Schematic representations of beam illumination at objective back focal plane (BFP) and sample planes for wide-field microscopy (single-beam illumination, **a**), 3D SIM (three-beam illumination, **b**) and four-beam SIM (a mirror opposite the sample is used to back-reflect the central beam, producing four-beam interference, **c**). Higher magnification illumination views at right show fine axial structure in four-beam SIM pattern, absent in 3D SIM or wide-field microscopy. **d**, Axial cross-sectional views of 100-nm beads, as imaged in wide-field microscopy (top), 3D SIM (middle) and four-beam SIM (bottom). **e**, Higher magnification views of bead highlighted by colored arrowheads in **d**, illustrating progressive improvement in axial resolution. Insets show magnitude of OTFs (k_x_/k_z_ plane) derived from images. **f**, Line profiles corresponding to bead images shown in **e**, taken along vertical green line in **e**. **g**, Quantification of lateral (blue) and axial (orange) FWHM for *n* = 102, 100 and 99 beads for wide-field microscopy, 3D SIM and four-beam SIM, respectively. See also Supplementary Table 1. Whiskers: maximum and minimum; center lines: medians; bounds of box: 75th and 25th percentiles; cross symbols: mean markers. Scale bars, 2 µm (**d**) and 500 nm (**e**); 1/200 nm^−1^ for Fourier transform insets in **e**. a.u., arbitrary units. Source data

**Extended Data Fig. 2:**
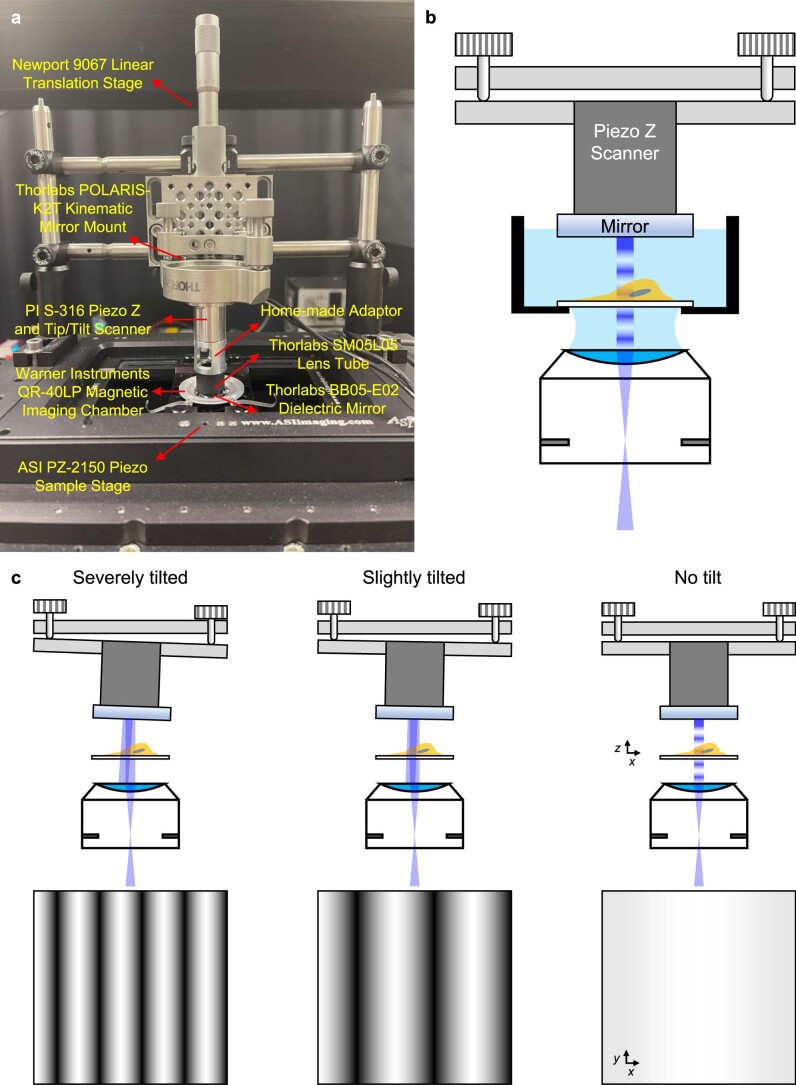
Mounting and aligning reflective mirror for four-beam SIM. **a**) Photograph of mounting scheme, indicating hardware mounts with vendor information. PI: Physik Instrumente. **b**) Schematic to accompany **a**), showing kinematic mirror mount, Piezo Z scanner, mirror, sample, and objective lens. Schematic is not to scale. **c**) Alignment of the reflected beam is achieved by manually adjusting the knobs on the kinematic mirror mount until tilt is minimized. This can be achieved by monitoring the autofluorescence from a dirty coverslip immersed in liquid; tilt is minimized when a flat intensity profile is achieved when translating the sample through focus, yielding a purely axial modulation. Top images show reflected beam at different tilts of mirror; bottom images show corresponding planar images of autofluorescence when lateral modulation is present (left, middle) versus mostly axial modulation (right). See also Supplementary Video 1 and Methods for more information.

We reasoned that placing a mirror directly opposite the sample by immersing it in the sample fluid would facilitate four-beam SIM (Fig. 1a–c), offering advantages over the previously proposed design^21^. (1) The mirror can be placed close (within 100 μm) to the sample, enabling the use of commonly available, short-coherence-length diode lasers. (2) The short optical path length from coverslip to mirror and back to coverslip implies that interferometric stability needs to be maintained only over this length scale. (3) Aberrations due to RI mismatch can be minimized. To demonstrate this method, we verified that commercially available 1.35 NA silicone oil or 1.27 NA water immersion objectives support four-beam SIM imaging (Supplementary Fig. 1); constructed a 3D SIM system that served as the base for our method, confirming the quality of our illumination pattern and raw data^24,25^ (Supplementary Figs. 2–7); and mounted and immersed a piezoelectrically controlled mirror directly over the sample, enabling four-beam SIM (Extended Data Fig. 2 and Supplementary Video 1).

**Extended Data Fig. 3:**
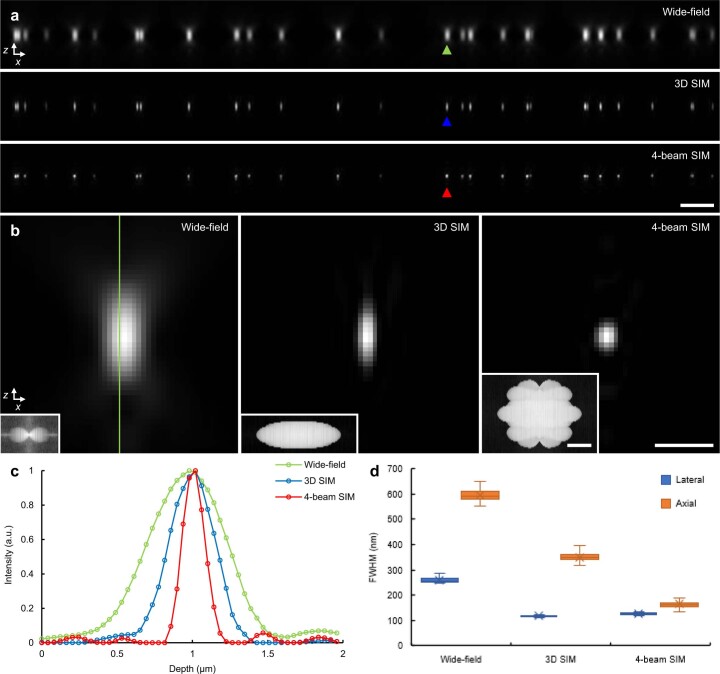
Estimating spatial resolution for 1.27 NA water lens. **a**) x-z cross sectional views of 100 nm yellow-green beads, as viewed with wide-field microscopy (top), 3D SIM (middle), and four-beam SIM (bottom). Scale bar: 2 µm. **b**) Higher magnification views of bead marked by green, blue, and red arrowheads in **a**). Insets show magnitude of corresponding Fourier transform. Scale bar: 500 nm, 200 nm^−1^ (inset). **c**) Line profiles taken along vertical line shown in **b**). **d**) Full width at half maximum (FWHM) analysis from *n* = 85, 81, 78 beads, showing lateral (blue) and axial (orange) values for the three methods. See also Fig. 1, Supplementary Table 1. Whiskers: maximum and minimum; centerlines: medians; bounds of box: 75th and 25th percentiles; cross symbols: mean markers. a.u., arbitrary units. Source data

We initially characterized our four-beam SIM by imaging 100-nm yellow-green beads using the 1.35 NA objective (Fig. 1d,e). Using 45.6% iodixanol^26^ to match the silicone oil RI, thereby minimizing spherical aberration^27^ and focal shift^28^, we collected 15 images (5 phases per orientation × 3 orientations) per plane and reconstructed image stacks similarly to 3D SIM (Methods). As expected, four-beam SIM maintained the ∼2-fold lateral resolution enhancement of 3D SIM over wide-field microscopy while offering ∼2-fold-better axial resolution than 3D SIM (Fig. 1f,g and Supplementary Table 1). We obtained similar results using the 1.27 NA water lens (Extended Data Fig. 3 and Supplementary Table 1). Bead imaging also highlighted the importance of keeping the illumination pattern maxima centered on the detection focal plane, as even a ∼40-nm shift resulted in axial ringing in the reconstructions (Supplementary Fig. 8a). We, thus, developed a bead-based feedback scheme that kept both focal plane and mirror stable to within 10–20 nm (Supplementary Figs. 8b,c and 9 and Methods).

### Near-isotropic super-resolution imaging

**Fig. 2:**
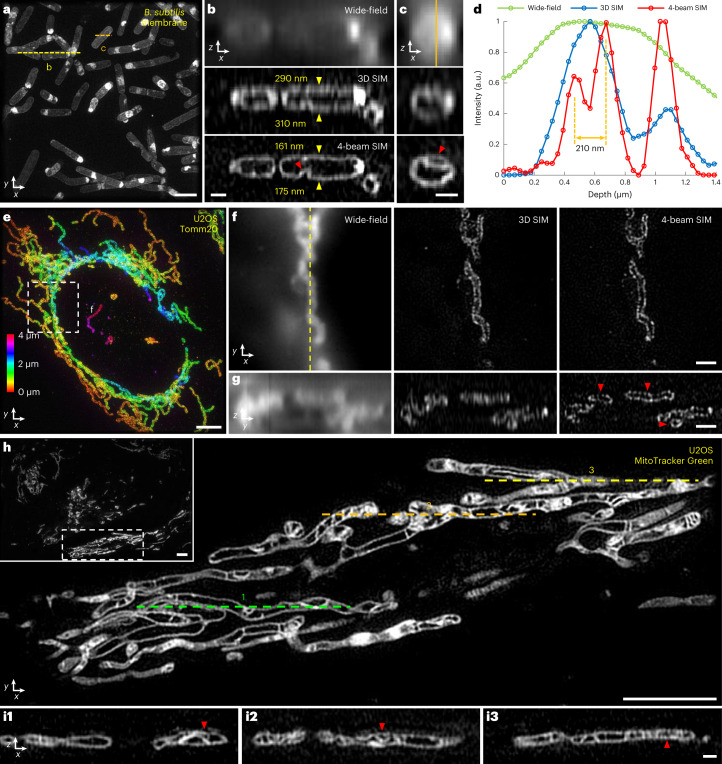
Four-beam SIM enables near-isotropic imaging of biological samples. **a**, Maximum intensity projection of live vegetative *B. subtilis* stained with CellBrite Fix 488, marking membranes, imaged in four-beam SIM. **b**,**c**, Axial views along yellow (**b**) and orange (**c**) dashed lines in **a**, comparing wide-field microscopy (top), 3D SIM (middle) and four-beam SIM (bottom). Yellow arrowheads in **b** highlight upper and lower cell membranes (numbered values indicate apparent membrane thickness); red arrowheads highlight membrane invaginations. See also Supplementary Video 2. **d**, Line profiles corresponding to vertical orange line in **c**. **e**, Maximum intensity projection of fixed U2OS cells labeled with Tomm20 primary and rabbit Alexa Fluor 488 secondary antibodies, marking outer mitochondrial membrane. Image is depth coded as indicated. Higher magnification lateral views (single planes) (**f**) corresponding to white dashed rectangle in **e** are shown, comparing wide-field microscopy (left), 3D SIM (middle) and four-beam SIM (right), in addition to corresponding axial views (**g**) taken across vertical yellow dashed line in **f**. Red arrowheads highlight void regions obscured in 3D SIM and wide-field microscopy. See also Supplementary Video 3. **h**, Overview (inset) and higher magnification view of single lateral plane of mitochondria labeled with MitoTracker Green FM in live U2OS cells, highlighting inner mitochondrial substructure. **i**, Axial cross-sections taken along green, orange and yellow dashed lines in **h** highlight fine substructure within mitochondria (red arrowheads). See also Supplementary Video 4. All data were acquired with 1.35 NA silicone immersion objective, with samples index-matched in 45.6% iodixanol. Scale bars, 2 µm (**a**); 500 nm (**b**,**c**,**i**); 4 µm (**e**,**h**); and 1 µm (**f**,**g**). a.u., arbitrary units. Source data

We next applied four-beam SIM to biological samples, first using the silicone oil lens on iodixanol RI-matched samples (Fig. 2). On ∼1-μm-thick, live, vegetative *Bacillus subtilis* stained with CellBrite Fix 488, four-beam SIM provided crisp lateral (Fig. 2a) and axial (Fig. 2b and Supplementary Video 2) views of cell membranes. Wide-field microscopy barely resolved cell membranes in axial views, which were better visualized in 3D SIM. However, four-beam SIM provided even more clarity (Fig. 2b), as seen visually and quantified from the apparent membrane thickness (∼160–175 nm). We observed fine membrane invaginations that appeared indistinct or badly blurred in 3D SIM (Fig. 2c,d), underscoring the ability of four-beam SIM to unveil axial detail otherwise masked by diffraction.

Next, we examined thicker eukaryotic samples spanning tens of microns in each lateral dimension (Fig. 2e–i). When performing wide-field imaging of fixed U2OS cells immunolabeled for Tomm20 (Fig. 2e), marking the outer mitochondrial membrane, diffraction obscured the inner mitochondrial space. By contrast, 3D SIM and four-beam SIM produced lateral views where such regions void of label were easily discerned (Fig. 2f). In axial views, however, only four-beam SIM could reliably resolve the void regions, as the poorer axial resolution of wide-field or 3D SIM artificially ‘filled in’ and distorted the inner mitochondrial space (Fig. 2g and Supplementary Video 3). We next stained living U2OS cells with MitoTracker Green FM, marking the internal mitochondrial space (Fig. 2h–i and Supplementary Video 4). Unlike 3D SIM^3^, four-beam SIM enabled visualization of fine mitochondrial substructure in both axial (Fig. 2i) and lateral views.

When using the 1.35 NA lens, we noticed that samples immersed in the RI-matched iodixanol solution displayed substantially more bleaching than in PBS (Supplementary Fig. 10). This fact, combined with the unknown effect of iodixanol on living samples at 45.6% composition and the awkward sample handling associated with such a viscous buffer, prompted us to abandon the silicone oil lens in favor of the 1.27 NA water lens.

Using this lens, we first verified that the resolution enhancement obtained on beads (Extended Data Fig. 3) extended to biological samples by imaging live, vegetative *B. subtilis* labeled with DivIVA-GFP (Supplementary Fig. 11a). DivIVA is known to assemble at nascent division sites^29^ and is thought to spatially regulate cell division. Both 3D SIM and four-beam SIM resolved the ‘double-ring’ DivIVA arrangement at the site of active division. However, the near-isotropic resolution of the latter also provided clearer axial views of the circularly shaped rings (Supplementary Fig. 11b and Supplementary Video 5), which otherwise assume a distorted ovoid appearance^29^.

**Fig. 3:**
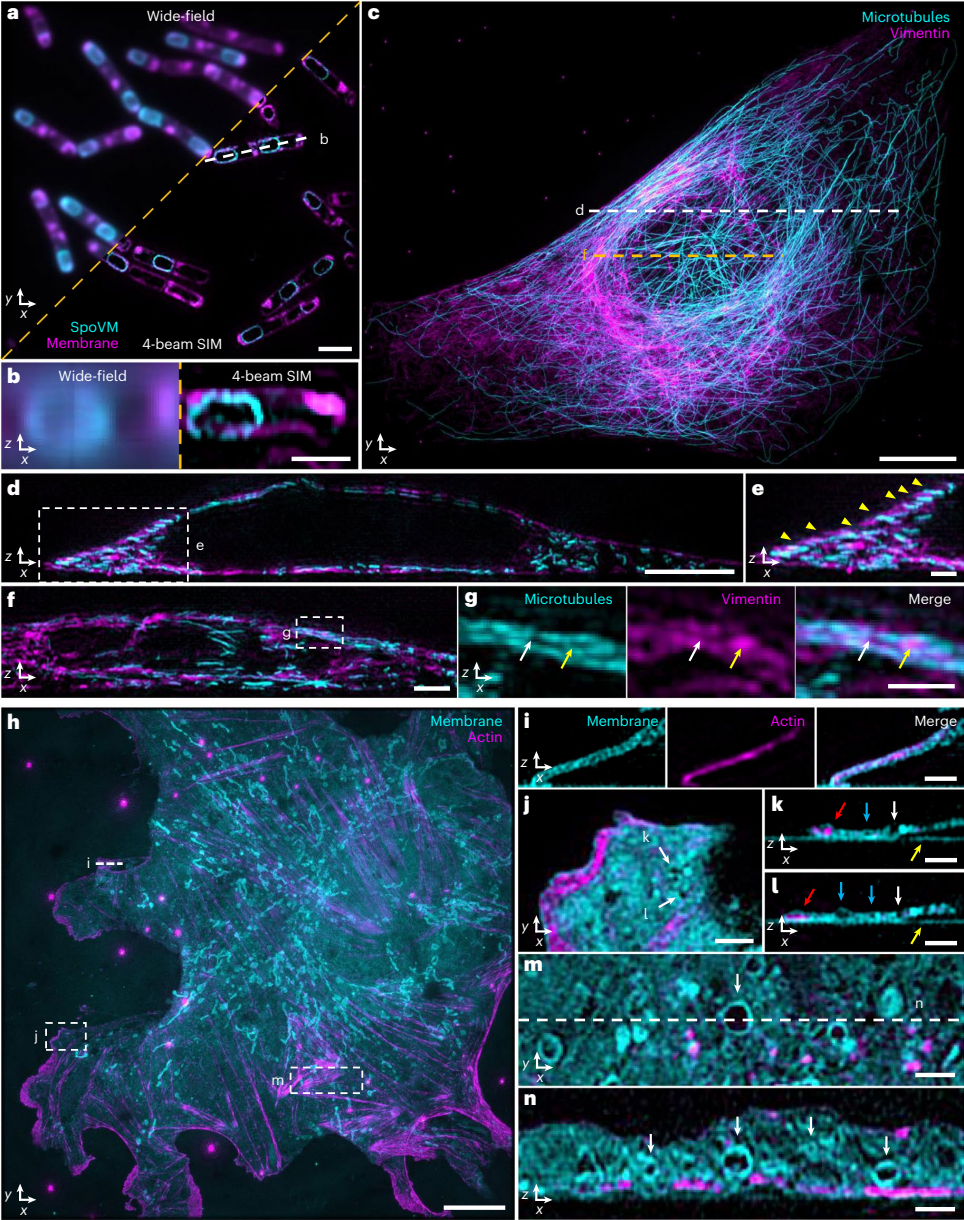
Near-isotropic imaging in two colors via four-beam SIM. **a**, Single lateral plane of live, sporulating *B. subtilis* with SpoVM-GFP label, marking spores (cyan), and CellBrite Fix 555 label, marking membrane (magenta). **b**, Axial view (single plane) along white dashed line in **a**. Images to the left of orange dashed line in **a** and **b** show wide-field images for comparison. **c**, Maximum intensity projection of fixed U2OS cells with Alexa Fluor 488-immunolabeled microtubules (cyan) and Alexa Fluor 594-immunolabeled labeled vimentin (magenta). **d**, Axial view corresponding to white dashed line in **c**. **e**, Higher magnification view of white dashed rectangular region in **d**, indicating apparent alternating stratification of microtubules and vimentin filaments (yellow arrowheads). Image is a maximum intensity projection over ten planes. **f**, Axial view corresponding to dashed orange line in **c**. Image is a maximum intensity projection over five planes. **g**, Higher magnification view of white dashed rectangular region in **f**, highlighting apparent ‘filling’ of inter-microtubule gaps by vimentin (arrows). Microtubule (left), vimentin (middle) and merged (right) images are shown. **h**, Maximum intensity projection image of fixed mouse LSECs with CellBrite Fix 488 label, marking membrane (cyan), and Alexa Fluor 568 phalloidin, marking actin filaments (magenta). See also Supplementary Video 6. **i**, Axial view corresponding to white dashed line in **h**, highlighting membrane signal encapsulating actin. Image is a maximum intensity projection over three planes. **j**, Higher magnification view of white dashed rectangular region in **h** with membrane pores highlighted (white arrows). **k**,**l**, Corresponding axial views to **j**, again highlighting the same pores (white arrows). Red arrows: actin encapsulated within membrane; blue arrows: void areas enclosed by membrane; yellow arrows: non-specific labeling of coverslip with membrane dye. **m**, Higher magnification view of dashed rectangular region in **h**, with accompanying cross-sectional view (**n**) corresponding to white dashed line in **m**, highlighting membrane-bound organelles (white arrows). Scale bars, 2 µm (**a**,**f**); 1 µm (**b**,**e**,**g**,**i**–**n**); 10 µm (**c**,**h**); and 5 µm (**d**). All images were acquired with four-beam SIM unless otherwise noted.

We next pursued dual-color imaging (Fig. 3). Illuminating the sample with distinct wavelengths (for example, 488 nm and 561 nm) produces different axial offsets between the illumination pattern maxima and detection focal plane. Ideally, this offset would be minimized for each illumination wavelength^24^, maximizing pattern contrast, optical sectioning and axial resolution. For four-beam SIM, minimizing this offset proved mandatory to minimize axial ringing in the reconstructions (Supplementary Fig. 12). We, thus, determined the optimal position of our camera for each illumination wavelength, translating it when switching color channels (Methods).

We first imaged live, sporulating *B. subtilis* cells expressing GFP-tagged SpoVM and additionally labeled with CellBrite Fix 555 (Fig. 3a). SpoVM preferentially binds to micron-scale convex membranes^30^, marking these surfaces to direct assembly of the proteinaceous ‘coat’ surrounding the developing spore^31^. Dual-color four-beam SIM enabled crisp visualization of SpoVM localized to mature spores within the surrounding membrane of the mother cell. Such detail was lost in wide-field imaging (Fig. 3b).

Second, we immunolabeled and imaged vimentin intermediate filaments and microtubules in fixed U2OS cells (Fig. 3c–g). Instead of co-localization (Fig. 3d), we observed an apparent ‘stratification’ of vimentin and microtubule fibers in the perinuclear area, underscoring the axial resolution of four-beam SIM (Fig. 3d,e). Intriguingly, we also observed examples in which local enrichments of vimentin appeared to fill the ‘gaps’ between microtubules (Fig. 3f,g), consistent with recent work showing that vimentin filaments can ‘template’ the microtubule network to stabilize cell polarity during migration^32^.

Finally, we imaged Alexa Fluor 568 phalloidin-labeled actin filaments and CellBrite Fix 488-labeled membranes in fixed mouse liver sinusoidal endothelial cells (LSECs) (Fig. 3h–n and Supplementary Video 6). Axial views revealed actin filaments encapsulated within fine membrane protrusions (likely filopodia; Fig. 3i) and smaller actin enrichments within the lamellar region (Fig. 3j–l). LSECs contain nanoscale pores (‘fenestra’) that filter material passing between blood and hepatocytes. Similarly to studies using 3D SIM^17^, we resolved vertically oriented pores traversing the extent of thin membrane regions as well as hollow regions fully encapsulated within the membrane (Fig. 3j–l). Elsewhere, in thicker cell regions, we observed many variably sized void regions, some just larger than our resolution limit and, others, microns in diameter (Fig. 3m,n). In almost all, the CellBrite marker was enriched around voids, suggesting that they represent membrane-bound organelles.

### Isotropic super-resolution imaging based on deep learning

When validating our four-beam SIM system, we imaged hundreds of cells. Two persistent challenges motivated us to consider alternate means of enhancing axial resolution. First, even though careful adjustment and stabilization of the instrument reliably yielded high quality reconstructions (Figs. 1–3, Extended Data Fig. 3 and Supplementary Fig. 11a,b), we occasionally observed ringing artifacts when imaging fine structures smaller than our resolution limit. Indeed, given that we mostly observed such artifacts when imaging microtubules at nuclear boundaries (Supplementary Fig. 11c–f), we suspect that slight variations in RI within the cell cause localized wavefront distortions that produce reconstruction artifacts.

Second, although we successfully imaged whole living cells (Figs. 2a–d,h–i and 3a,b and Supplementary Fig. 11a,b), volumetric time-lapse (‘4D’) imaging proved challenging. Even when using the bright and photostable dye Potomac Gold^33^ to ubiquitously label cell membranes, we found that phototoxicity^34^ limited experiment duration (Supplementary Fig. 11g,h and Supplementary Video 7). In hindsight, this is unsurprising: imaging even a modestly sized 4-μm-thick volume with four-beam SIM entails 1,000 raw images (assuming 15 images per plane and our 60-nm axial sampling interval) with each raw image capture illuminating most of the cell volume.

Given that 3D SIM introduces less dose than four-beam SIM, is more robust to wavefront distortions (Supplementary Fig. 11d) and can enable sustained 4D imaging^3^, we considered computational strategies for improving the axial resolution of 3D SIM without introducing additional illumination dose. As deep learning can enhance spatial resolution^35–39^, we evaluated a method that improves axial resolution by (1) blurring and downsampling lateral views to resemble lower-resolution axial views and (2) learning to reverse this degradation based on the higher-resolution lateral view ground truth^35,40^. The strength of this approach is that the training data itself contain the ground truth, although a key assumption is that the structures of interest appear similar regardless of viewing direction.

**Fig. 4:**
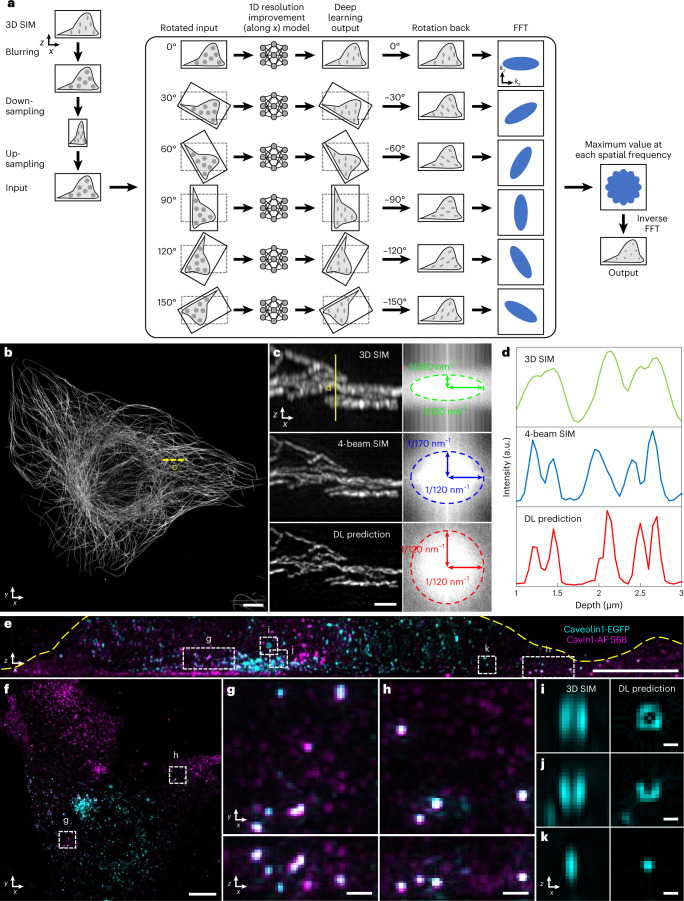
Deep learning for axial resolution enhancement. **a**, Schematic of deep learning process. 3D SIM image volumes are blurred, downsampled and upsampled (each along the lateral *x* direction) to render isotropic, low-resolution input data (resolution equivalent to axial resolution in 3D SIM). This input (1) is rotated in 30° increments; (2) each rotation is passed through a deep learning model to improve resolution along the *x* direction, (3) rotated back to the original frame and (4) Fourier transformed; (5) the maximum value, taken over all rotations, at each spatial frequency was recorded and, finally, (6) inverse Fourier transformed to yield an output prediction with improved isotropic resolution. See also Supplementary Fig. 14 and Methods. **b**, Alexa Fluor 488-immunolabeled microtubules in a fixed U2OS cell. Maximum intensity projection of deep learning prediction is shown. **c**, Left: higher magnification axial view indicated by yellow dashed line in **b**, comparing 3D SIM (top), four-beam SIM (middle) and deep learning prediction (bottom). Images are generated by computing maximum intensity projection over 20 pixels in the *y* direction. Right: magnitudes of Fourier transforms corresponding to images at left, with indicated spatial frequencies bounding the major and minor ellipse axes. **d**, Line profiles corresponding to yellow solid line in **c**. **e**,**f**, Fixed MEF with caveolin-1 EGFP with GFP booster (cyan) and Alexa Fluor 568-immunolabeled cavin-1 (magenta). Axial (**e**) and lateral (**f**) maximum intensity projections of deep learning predictions are shown. Yellow dashed line in **e** has been added to better delineate the cell boundary. **g**,**h**, Higher magnification views of dashed rectangular regions in **e** and **f** with lateral (top) and axial (bottom) projections indicated. **i**–**k**, Higher magnification axial views of regions indicated in **e**, comparing 3D SIM input (left) to deep learning prediction (right). See also Supplementary Fig. 15. Scale bars, 5 µm (**b**,**e**,**f**); 1 µm (**c**); 500 nm (**g**,**h**); and 200 nm (**i**–**k**). a.u., arbitrary units; DL, deep learning. Source data

When evaluating the method on randomly oriented simulated structures that had been blurred with an anisotropic point spread function (PSF), we were able to restore the structures to isotropic resolution (Supplementary Fig. 13a,b). However, when we attempted to restore 3D SIM data, although the network improved axial resolution for some structures (Supplementary Fig. 13c), it also artificially distorted the shape of others or even lost them (Supplementary Fig. 13d–h), likely because axial specimen views looked quite different than the lateral specimen views that the network was trained on. We reasoned that performance would improve if the network was directly exposed to axial information during the training process. We, thus, presented the network with axial (*x–z*) 3D SIM views that had been blurred and downsampled to yield data with isotropic resolution equivalent to the axial resolution of 3D SIM, and then we trained the network to reverse the degradation along the lateral direction (Supplementary Fig. 14 and Methods). Motivated by a related pipeline^41^, we applied the trained network to six digitally rotated views of unseen, similarly degraded 3D SIM data, enabling the improvement of 1D resolution along arbitrary directions. Subsequent fusion of all six resolution-enhanced views^42^ yielded a final prediction with isotropic resolution (Fig. 4a and Methods).

**Extended Data Fig. 4:**
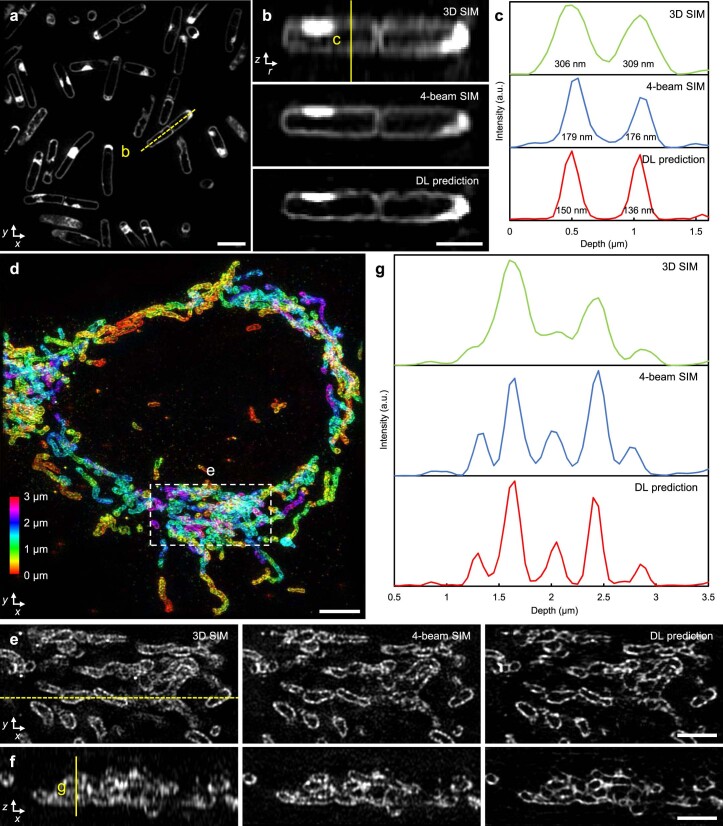
Images of additional samples, comparing deep learning isotropization output to 3D SIM and four-beam SIM. **a**) DL prediction images of live *B. subtilis* stained for membrane (CellBrite Fix 488). **b**) Axial views along dashed yellow line shown in **a**), comparing 3D SIM (top), four-beam SIM (middle), and deep learning (DL) prediction based on 3D SIM input. **c**) Line profiles corresponding to yellow vertical line in **b**). Full width at half maximum (FWHM) values under each peak are indicated. **d**) Depth-color coded images of immunolabeled Tomm20 in fixed U2OS cells. Maximum intensity projection of DL prediction is shown. **e**) Higher magnification views of dashed region in **d**), with lateral (top) and corresponding axial views **f**) (corresponding to dashed yellow line in lateral view) comparing 3D SIM (left), four-beam SIM (middle) and DL prediction (right). **g**) Line profiles along yellow vertical line in **f**). Scale bars: 2 µm **a, e, f**), 1 µm **b**), 4 µm **d**). a.u., arbitrary units. Source data

Comparing images of the same sample produced by 3D SIM, four-beam SIM and our deep learning prediction (Fig. 4b–d) facilitated validation of the method. For example, when inspecting immunolabeled microtubules in fixed U2OS cells (Fig. 4b), although all three methods offered similar lateral resolution (Fig. 4c), fine axial features blurred in 3D SIM were resolved with four-beam SIM and the network prediction, which showed close visual and quantitative (Fig. 4c,d) agreement. We obtained similar results on membrane-stained, live *B. subtilis* and immunolabeled Tomm20 in fixed U2OS cells (Extended Data Fig. 4) and verified the resolution improvement on additional samples using a modified decorrelation analysis method (Supplementary Table 2)^37,43^.

Next, we performed two-color imaging of caveolin-1 and cavin-1, components of the caveolar coat. Caveolae are 70–100-nm-diameter membrane invaginations that can detach from the plasma membrane and move through the cytoplasm, playing key roles in lipid metabolism and trafficking^44^. We fixed mouse embryonic fibroblasts (MEFs) expressing caveolin-1-EGFP and additionally immunolabeled cavin-1 with Alexa Fluor 568, performed 3D SIM imaging and then applied our network to the 3D SIM images (Fig. 4e–k). Caveolin-1 and cavin-1 labels mostly marked distinct caveolae pools (Fig. 4e,f), although we also observed a smaller pool of caveolae puncta with co-localized signal (Fig. 4g,h). Unlike the cavin-1 signal, which mostly decorated structures sized at or below our resolution limit, caveolin-1 labeled a more heterogenous pool of caveolae (Fig. 4i–k). Hints of such heterogeneity existed in the input 3D SIM data but were obscured by diffraction. By contrast, the network prediction appeared to resolve ring-shaped structures (Fig. 4i), partial rings (Fig. 4j) and spherical puncta (Fig. 4k). We also found that caveolin-1 localized to larger ring-shaped structures of varying size, possibly lipid droplets (Supplementary Fig. 15).

Using an inappropriate deep learning model can introduce artifacts and/or reduce axial resolution. We investigated this point by using ‘mismatched’ training data—for example, a model with training data derived from microtubule samples and applied to Tomm20 mitochondrial data and a model trained on Tomm20 mitochondrial samples and applied to microtubule data (Supplementary Fig. 16). Although the gross morphology of the samples is maintained, closer examination revealed that such mismatches produce suboptimal predictions.

### Multi-step deep learning enables 4D super-resolution imaging

**Fig. 5:**
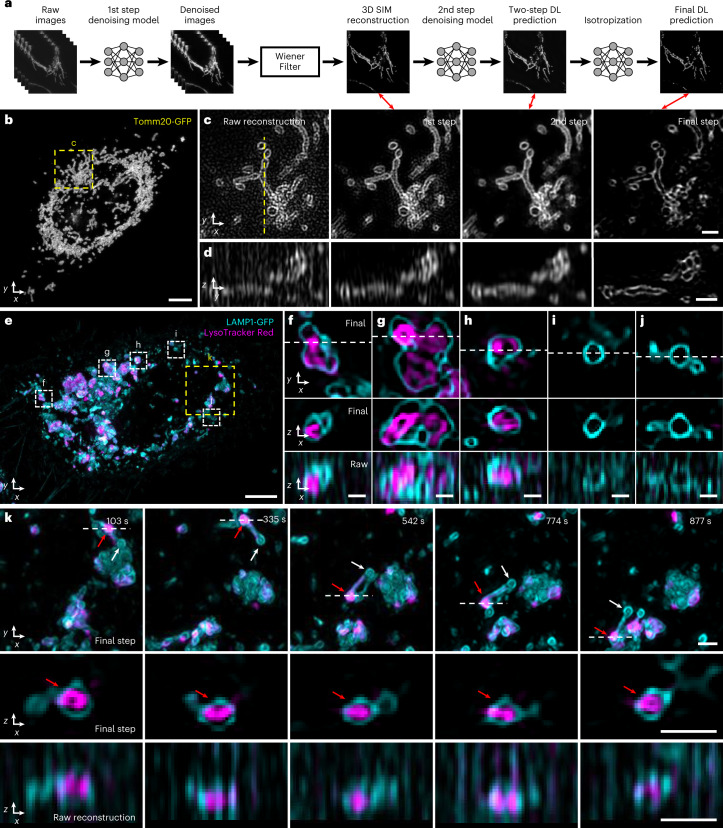
Denoising and axial resolution enhancement facilitate 4D super-resolution imaging with isotropic resolution. **a**, Schematic illustrating workflow for applying deep learning to raw input data. Sets of raw images (5 phases × 3 orientations) are denoised and combined with a generalized Wiener filter; and the resulting 3D SIM reconstruction is denoised and passed through our axial resolution enhancement workflow (Fig. 4a) to yield an isotropic, denoised, super-resolution prediction. See also Fig. 4a and Supplementary Figs. 14 and 17. **b**, Maximum intensity projection of final prediction for Tomm20-GFP label in a live U2OS cell, 25th timepoint from a 50-timepoint volumetric series. See also Supplementary Video 8. **c**, Single lateral plane corresponding to yellow dashed rectangular region in **b**, illustrating progressive improvement from 3D SIM reconstruction based on raw input data; after the first denoising model and Wiener filter; after applying the second denoising model; and after the isotropization model. Double-headed red arrows show corresponding steps in schematic **a**. **d**, As in **b** but for axial plane indicated by yellow dashed line in **c**. **e**, Maximum intensity projection of final prediction for live U2OS cells expressing lysosomal marker LAMP1-GFP (cyan) and additionally labeled with LysoTracker Red to mark the lysosome interior (magenta). First timepoint from a 60-timepoint volumetric series is shown. See also Supplementary Video 10. **f**–**j**, Higher magnification views of white dashed rectangular regions in **e**, illustrating lateral views (top), axial views along white dashed lines in lateral views (middle) and comparative axial views from 3D SIM reconstructions (bottom, ‘Raw’). **k**, Higher magnification view of yellow dashed rectangular region, emphasizing dynamics at selected timepoints. See also Supplementary Video 12. Top: lateral views, red arrow emphasizes lysosomal subregion filled by LysoTracker Red dye versus white arrow, indicating unfilled region; middle: axial view corresponding to white dashed lines; bottom: comparative 3D SIM axial view. Scale bars, 5 µm (**b**,**e**); 1 µm (**c**,**d**,**k**); and 500 nm (**f**–**j**). DL, deep learning; s, seconds.

One of the easiest ways to extend imaging duration in fluorescence microscopy is to lower the illumination intensity. This approach is ultimately limited by signal-to-noise ratio (SNR) in the raw data, a particularly important constraint in conventional 3D SIM reconstruction, which is highly susceptible to noise^45^. In response to this challenge, several recent studies employed deep learning to denoise SIM data^38,46–48^. Motivated by this work, we developed a multi-step denoising approach (Fig. 5a, Supplementary Fig. 17a and Methods). First, we gathered matched pairs of low and high SNR volumes (∼10-fold difference in illumination intensity), training a residual channel attention network (3D RCAN^37^) to denoise low SNR volumes that would otherwise produce unacceptably noisy 3D SIM reconstructions. Next, we applied a generalized Wiener filter to produce an intermediate 3D SIM reconstruction from the denoised low SNR volumes. Finally, we trained a second 3D RCAN to additionally denoise the intermediate reconstruction, further reducing patterned noise artifacts (Fig. 5b–d and Supplementary Fig. 17b,c). This procedure provided reconstructions that were visually and quantitatively superior to those produced by all other strategies that we tested (Supplementary Figs. 18 and 19 and Supplementary Tables 3 and 4), including using only the first 3D RCAN and subsequent Wiener filter; modifying the RCAN to incorporate all 15 low SNR input volumes to directly (without a Wiener filter) predict the 3D SIM reconstruction; using DenseDeconNet^49^, a different type of network; or eliminating the Wiener filter between the sequential 3D RCAN networks. In this work, we built multi-step denoising models for outer mitochondrial membranes (Tomm20), lysosomal membranes (LAMP1), interior lysosomal markers (LysoTracker Red) and microtubules.

Our multi-step denoising pipeline produced high-quality 3D SIM predictions that could be input to our axial resolution enhancement method (Fig. 5a), thereby producing high SNR reconstructions with ∼120-nm isotropic resolution from low SNR input. The improvement in SNR and resolution offered by deep learning was particularly striking when visualizing subcellular organelles and their dynamics. For example, by lowering the illumination intensity to ∼0.5 W/cm^2^, we could perform 3D SIM imaging of EGFP-Tomm20 in live U2OS cells (Fig. 5b and Supplementary Video 8) for 50 volumes without substantial photobleaching or obvious phototoxicity. Performing the direct 3D SIM reconstruction on the low SNR raw data revealed the expected Tomm20 signal at the outer mitochondrial membrane in lateral views, although the signal was contaminated with patterned noise (Fig. 5c) and obscured in axial views (Fig. 5d). Denoising through successive 3D RCANs progressively improved SNR, although only the final step provided an axial clarity commensurate with lateral views (Fig. 5d and Supplementary Video 8). We observed similar improvements when imaging lysosomal dynamics with an EGFP-LAMP1 marker in live U2OS cells (Supplementary Video 9).

To demonstrate the potential of our deep learning pipeline for live dual-color imaging, we performed volumetric imaging over 60 timepoints of U2OS cells expressing EGFP-LAMP1, marking lysosomal membranes, and additionally labeled with LysoTracker Red dye, which labeled the interior of lysosomes (Fig. 5e–k and Supplementary Videos 10 and 11). Although some lysosomes appeared as ‘textbook’ discrete vesicular structures (Fig. 5i,j), our reconstructions revealed considerable structural heterogeneity, with some lysosomes assuming a ‘multi-bud’ structure (Fig. 5f,g) and others appearing tubular (Fig. 5k). LysoTracker Red localized to the interior of many, but not all, lysosomes. In some cases, LysoTracker Red preferentially localized to different membrane-bound regions even within a single lysosomal structure (Fig. 5f,g,k). Such compartmentalized staining appeared stable over our ∼25-minute recording, suggesting limited turnover of the dye despite rapid dynamics of the parent structure (Fig. 5k and Supplementary Video 12). Given that LysoTracker Red is known to stain acidic organelles, perhaps this result reflects underlying pH differences between and even within lysosomes. Such fine structural details were obscured in axial views of 3D SIM reconstructions derived from the raw data.

**Fig. 6:**
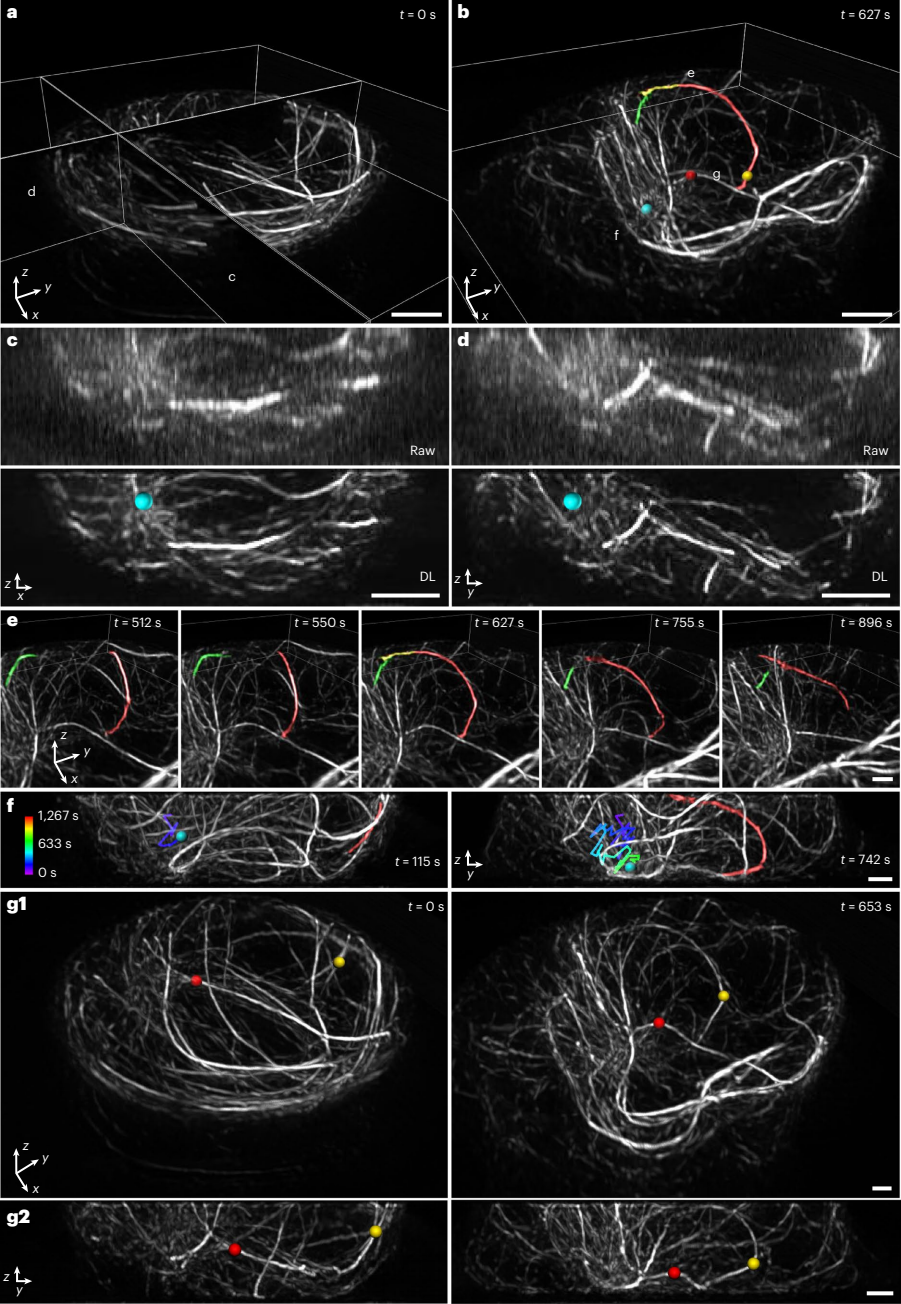
Denoising and axial resolution enhancement unveil rich microtubule dynamics within a living immune cell. **a**,**b**, Selected volumetric reconstructions of Jurkat T cells expressing EMTB 3× GFP are shown from 100-timepoint series (volumes recorded every 12.8 seconds) in perspective views. In **b**, the MTOC (cyan sphere), overlapping microtubules (red, yellow, green) and buckling microtubules (red and yellow spheres) are shown. **c**,**d**, Comparative axial views (maximum intensity projections over 2-µm thickness in *y*) of 3D SIM reconstructions (‘Raw’, upper row) and deep learning output (‘DL’, bottom row) corresponding to planes in **a**. Position of MTOC is indicated (cyan sphere). **e**, Selected timepoints corresponding to subregion marked in **b**, emphasizing two microtubule filaments that are initially separated (*t* = 512 seconds and 550 seconds), merge over the nucleus (*t* = 627 seconds) and separate again (*t* = 755 seconds and 896 seconds). See also Supplementary Video 13. **f**, Axial views (projections over 8-µm thickness) that emphasize correlated, inward movement of MTOC (cyan sphere; previous trajectory temporally coded as indicated in color bar) and microtubule filament (red; the same filament shown in **b** and **e**). See also Supplementary Video 14. **g**, Lateral (**g1**) and axial (**g2**) views emphasizing buckling of two microtubule filaments (red and yellow spheres, as shown in **b**). Pre-buckling: left columns; post-buckling: right columns. Both **g1** and **g2** are projections over 8-µm thickness. See also Supplementary Video 15. Scale bars, 2 µm (**a**–**d**) and 1 µm (**e**–**g**). DL, deep learning; s, seconds.

Finally, we investigated microtubule dynamics in Jurkat T cells as they were activated and spread on anti-CD3 coverslips (Fig. 6 and Supplementary Videos 13–16). This system has been used extensively to study the cytoskeletal remodeling that takes place during the early stages of immunological synapse formation, including actin ring formation and centrosome polarization^50–52^. By lowering the illumination intensity sufficiently, we recorded a 3D SIM time series spanning 100 volumes (1 volume every 12.8 seconds) without substantially bleaching our EMTB 3× GFP microtubule marker or introducing noticeable phototoxicity. This ∼21-minute-duration recording proved long enough to observe substantial cytoskeletal remodeling, particularly after applying our denoising and axial resolution enhancement pipeline (Fig. 6a,b). The deep learning prediction revealed finer detail and many more filaments than 3D SIM reconstructions based on the raw data, which were degraded by noise and poor axial resolution (Fig. 6c,d). The improvement in image quality facilitated inspection of the rapidly remodeling microtubule network, proving particularly helpful in elucidating the interplay of distinct cytoskeletal elements occurring perpendicular to the coverslip surface. For example, we segmented two microtubule filaments (Fig. 6b) that appeared to grow toward each other, merging and encircling the nucleus before disassociating (Fig. 6e and Supplementary Video 13). We also identified the microtubule organizing center (MTOC), tracking its ‘downward and inward’ movement from its original peripheral location toward the coverslip. Centrosome polarization in T cells has been described as biphasic and driven by the association of microtubule bundles with molecular motors (dynein) at the synapse^52,53^. Intriguingly, MTOC repositioning correlated closely with the inward movement of one of the segmented microtubule bundles (Fig. 6f and Supplementary Video 14), suggesting close physical coupling between different parts of the network, perhaps resulting from the coordinated action of microtubule-associated motors. We also observed numerous ‘buckling’ events of individual microtubule filaments (Fig. 6g, Supplementary Fig. 20 and Supplementary Videos 15 and 16), presumably also the result of active force generation by molecular motors^50^.

## Discussion

Improving the axial resolution of fluorescence microscopy can directly translate into new biological insights, yet most methods have focused on improving lateral resolution. In this study, we addressed this issue by improving the axial resolution of 3D SIM^1^, a broadly used super-resolution method well suited for studying single cells. First, we showed that adding a mirror to a 3D SIM system enables near-isotropic spatial resolution. The main advantage of this ‘physics-based’ four-beam SIM method is that it does not require information about the sample. By contrast, the second computational method can be applied to 3D SIM systems without hardware modification, instead embedding information about the sample into a series of neural networks, which can predict a denoised image reconstruction with isotropic spatial resolution. Although these techniques provide distinct means for axial resolution enhancement, they could be combined—for example, denoising the input to four-beam SIM so that illumination intensity may be lowered, thereby reducing phototoxicity and improving the potential for 4D imaging.

Both methods can be further improved. The four-beam SIM requires active drift correction and precise alignment of the illumination pattern. Although our bead-based algorithm (Supplementary Fig. 9) met both requirements, hardware-based correction^54^ would provide faster feedback, possibly obviating the need to image beads and, thus, lowering the total illumination dose imparted to the sample. For applications in which the axial view alone is sufficient, collecting only five phases (that is, one orientation) would provide optical sectioning and axial resolution enhancement, improving speed (and reducing dose) by three-fold. Also, for multi-color applications, the technique could benefit by implementing independent detection paths for each color, bypassing the time-consuming need to realign the detection path as manually implemented here.

The multi-step deep learning pipeline is currently time-consuming, requiring the collection of ∼50 volumetric pairs per network and ∼12 hours for training all networks. Once trained, application of the networks is faster, requiring ∼5 minutes per volume (each ∼500 × 500 × 80 voxels). Given continued progress in deep learning, improved networks with fewer parameters^55^ are likely to substantially shrink these times in the future. In our multi-step approach, we used the 15 raw image volumes required for traditional 3D SIM reconstruction as input to our networks. One route to faster and more gentle imaging is to use fewer input images (for example, fewer phases or coarser axial sampling), although the quality of such reconstructions is currently worse than obtained using the entire dataset^48^. Perhaps the largest caveats with any deep learning method are that the quality of the prediction is tied to the quality of the training data and that generalizability to data unlike that of the training set remains questionable. Finally, we note that the spatial resolution of both the four-beam and deep learning approaches may be improved by incorporating photoswitching^56^, albeit with accompanying reduction in temporal resolution and increase in illumination dose.

Nevertheless, in their current form, the methods we present outperform recent state-of-the-art SIM implementations. Relative to a recent implementation of lattice light-sheet microscopy (LLSM) employing structured illumination for lateral resolution enhancement and coherent detection for improved axial resolution (3D-iLLS)^57^, our techniques offer a 2–3-fold improvement in acquisition speed, a 2–3-fold improvement in volumetric resolution, a ∼40-fold improvement in imaging volume size, an order of magnitude more timepoints (using deep learning) and less susceptibility to reconstruction artifacts. Relative to live cell PA-NL SIM LLSM^58^, our techniques offer similar speed and resolution, ∼1.5-fold improvement in imaging volume size and 2.5–5-fold more timepoints (using deep learning) and do not require a photoswitchable fluorophore or a multi-objective imaging system. In addition to the gains on conventionally prepared samples illustrated here, our methods may improve SIM reconstructions on samples prepared for correlative super-resolution fluorescence and electron microscopy^59^, which currently suffer from poor axial resolution. Finally, although we focused here on improving the performance of 3D SIM, our deep learning methods may also prove useful in improving the axial resolution of other 3D imaging techniques (for example, confocal microscopy, stimulated emission depletion (STED) microscopy and instant SIM^60^), with the caveat that current neural networks^37,38,41^ appear able to produce 2–3× resolution enhancement at best.

## Methods

### Simulations of OTF support

The simulated 2D OTF supports (Extended Data Fig. 1 and Supplementary Fig. 1) were assembled based on imaging parameters and geometric considerations. The 3D OTF support of the wide-field microscope is a toroidal solid, whose 2D analog in the *k*_*r*_, *k*_*z*_ plane consists of the area enclosed by four arcs (Extended Data Fig. 1a). The position and extent of these arcs in the spatial frequency domain were computed by considering the detection NA and emission wavelength of the wide-field imaging system. The lateral and axial toroidal extents of the OTFs were determined as ${\textstyle{{2NA} \over \lambda }}$ and ${\textstyle{{\left( {1 - cos\alpha } \right) \cdot n} \over \lambda }}$, respectively, where NA is the numerical aperture of the objective lens for emission wavelength λ; *α* is the light-gathering half-angle of the objective lens; and n is the RI of the sample medium. The 3D SIM OTF was created by placing wide-field OTFs at each 3D SIM illumination frequency component (Extended Data Fig. 1b). The standing-wave microscope OTF support consists of the wide-field OTF and two duplicates along the *k*_*z*_ axis, positioned at the spatial frequencies of the standing wave determined by the excitation wavelength and RI (Extended Data Fig. 1c). The OTF support for four-beam SIM can be similarly derived by considering the area enclosed by wide-field OTFs placed at the seven 3D SIM illumination frequency components, the standing-wave spatial frequencies and four additional frequency components determined by interference of the reflected beam with the two side beams (Extended Data Fig. 1e). Finally, as previously described^20^, the I^5^S OTF support is determined by considering the area enclosed by the placing the I^2^M OTF^61^ at each of the 19 illumination components produced in I^5^S (Extended Data Fig. 1d). 3D OTF supports were simulated by converting each 2D coordinate in the *k*_*r*_, *k*_*z*_ plane to a corresponding set of 3D spherical coordinates (Supplementary Fig. 1).

### Home-built 3D SIM system

Our 3D SIM optical layout (Supplementary Fig. 2) was inspired by previous designs^1,3,4^. Two linearly polarized lasers (488 nm and 561 nm, Coherent, Sapphire 488 LP-300 mW and Sapphire 561 LP-200 mW) were combined via a 3-mm-thick dichroic mirror (DM1: Semrock, Di03-R405/488/532/635-t3-25×36) and passed through an acousto-optic tunable filter (AOTF, AA Opto-Electronic, AOTFnC-400.650-TN) for rapid shuttering and intensity control. Illumination power was measured after the objective, and the computed intensity at the sample plane varied between 0.5 W/cm^2^ and 25 W/cm^2^ based on a circularly illuminated area with diameter of 90 µm. The first-order beam exiting the AOTF was selected (the zero-order beam was blocked by a beam dump (BD)), expanded (L1 and L2; Thorlabs, TRH127-020-A-ML and ACT508-400-A-ML) and spatially filtered by a pinhole (P; Thorlabs, P30K), resulting in a beam with 15-mm 1/*e*^2^ diameter. The excitation beams were then redirected onto a phase-only nematic spatial light modulator (SLM; Meadowlark Optics, MSP1920-400-800-HSP8) at near-normal incidence (<6°). A half-wave plate (HWP; Thorlabs, AHWP10M-600) positioned before the spatial filter was used to adjust the direction of linear polarization, aligning it for maximum phase modulation by the SLM, thereby ensuring high contrast for the 15 patterns used in 3D SIM. An adjustable iris (Thorlabs, SM1D12) between the beam expander and the SLM was set to 10-mm diameter, slightly shorter than the short edge of the SLM active area (10.6 mm). Expanding the beam while underfilling the SLM improves illumination uniformity while avoiding diffraction effects from the SLM boundary.

Lens L3 was positioned at one focal length after the SLM, producing a Fourier image of the illumination pattern at its focus. A pinhole mask (PM) placed at this plane served to filter out unwanted illumination orders due to SLM pitch and pattern pixelization. The spatially filtered beams emerging from the PM were imaged via another pair of relay lenses (lens pair L4 and L5 placed in 4f configuration) onto the back focal plane (BFP) of the objective lens. For each pattern orientation, the coherent 0th and ±1st order beams were collimated by the objective and interfered at the sample plane to form the 3D SIM illumination pattern. A liquid crystal polarization rotator (LCPR; Meadowlark Optics, LPR-200-0525-ACHR, achromatic) was used for rapid rotation of the polarization state after SLM, producing s-polarized illumination at the sample and, thus, high illumination pattern contrast there. If using a silicone oil objective lens (Olympus, UPLSAPO ×100/1.35 NA), we used *f*_3_ = 250 mm, *f*_4_ = 300 mm and *f*_5_ = 250 mm (Thorlabs, AC508-250-A-ML and AC508-300-A-ML), producing a demagnification of 115.7 from the SLM to the sample plane, given that *f*_*obj*_ = 1.8 mm. When using a water objective lens (Nikon, CFI SR Plan Apo ×60/1.27 NA), we use *f*_3_ = 300 mm, *f*_4_ = 250 mm and *f*_5_ = 250 mm, producing a demagnification of 90.1 from the SLM to the sample plane given that *f*_*obj*_ = 3.33 mm. In both cases, the usable field of view (FOV) was at least 90 µm × 90 µm. In addition to FOV, several other design criteria informed our choice of *f*_4_–*f*_5_. First, we picked *f*_3_ to be sufficiently large (>200 mm) to (1) ensure sufficient room for near-normal incidence of the illumination beam at the SLM and (2) clearly separate the Fourier components of the illumination at the PM, allowing clean filtering of these components relative to background orders. Similarly, we picked *f*_5_ to be long enough (>200 mm) to accommodate a turning mirror and the dichroic mirror.

Fluorescence was isolated post-objective via a dichroic mirror (DM2; Semrock, Di03-R488/561-t3-25×36) and imaged to a scientific complementary metal-oxide semiconductor (sCMOS) detector (PCO, Edge 4.2HQ) mounted on a multi-axis translation stage (Thorlabs, XR25P-K1) and a vertical travel platform (Thorlabs, L490) via tube lens L6. Emission filters mounted in a filter wheel (FW; Applied Scientific Instrumentation, FW-1000) served to further reject illumination light and select appropriate spectral bands. In this work, two bandpass emission filters (Semrock, FF03-525/50-25 and FF02-617/73-25) and one notch emission filter (Semrock, NF03-405/488/561/635E-25) were used, depending on the sample. Bandpass filters were used when imaging yellow-green beads, red beads and all biological samples to avoid crosstalk between spectral bands. The notch filter was used only when imaging orange beads to align the four-beam SIM system for two-color applications. When using the 1.35 NA silicone oil objective lens, we chose *f*_6_ = 165 mm (Thorlabs, TTL165-A). When using the 1.27 NA water objective, we chose *f*_6_ = 265 mm (Applied Scientific Instrumentation, C60-TUBE-265D). The resulting image pixel sizes for the silicone oil lens were 70.9 nm (×91.7 magnification from sample to camera) and 81.8 nm (×79.5 magnification from sample to camera) for the water lens. In both cases, the image pixel sizes were smaller than the Nyquist limit.

Sample and objective were held in a modular microscope frame with a motorized *x*–*y* stage (Applied Scientific Instrumentation, RAMM and MS-2000 XYZ Automated Stage) used for lateral sample positioning and coarse focusing. A *z* piezo stage (Applied Scientific Instrumentation, PZ-2150, 150-µm axial travel) attached to the stage was used to provide precise axial sample positioning (125-nm step size for 3D SIM and 60-nm step size for four-beam SIM to ensure Nyquist sampling in *z*). Samples were deposited on high-precision 25-mm coverslips (Thorlabs, CG15XH) that were mounted in a magnetic imaging chamber (Warner Instruments, QR-40LP) filled with imaging medium. The chamber was placed into a stage insert (Applied Scientific Instrumentation, I-3091 universal insert) mounted to the piezo stage (Extended Data Fig. 2a).

### 3D SIM pattern generation

As in previous 3D SIM systems, we used the SLM as a binary phase grating (each pixel producing a phase retardance of 0 or π radians) to generate periodic illumination patterns at the sample plane. To generate appropriate patterns, we carefully considered how to implement (1) the desired pattern orientations, (2) the desired line spacing (grating period) in each pattern, (3) the duty cycle appropriate for each pattern and (4) the relative 2π/5 phase shifts between each of the five patterns required at each orientation.

First, 3D SIM typically uses patterns with three orientations spaced 60° apart to (1) achieve near-isotropic lateral resolution and (2) fill in the ‘missing cone’ of axial spatial frequencies, thereby providing optical sectioning. In the pixelated coordinate system of the SLM (Supplementary Fig. 3a), we found it convenient to define a vector $\overrightarrow A$, described by integer components (*A*_*x*_, *A*_*y*_), to specify pattern orientation. In this work, we chose $\overrightarrow A = \left( {2, - 11} \right)$, (14,−5) and (13,11) as the three pattern orientations, which correspond to 10.3°, 70.3° and 130.2°. This choice allowed us to pick a grating period with a non-integer value, unlike orientations at 0° or 90°, which would restrict the grating period to integer values. We also found that 45° and 135° orientations should be avoided, as they caused many additional orders between the zero and first orders at the Fourier plane, making the filtering at the PM less efficient.

Second, 3D SIM uses three tightly focused beams at the BFP of the objective lens to produce the illumination pattern. The positions of the two side beams are typically located at 90–95% of the pupil radius. Using a higher radius (>95% of the pupil) decreases the amplitude of the highest lateral illumination spatial frequency to the point that it is difficult to detect, complicating conventional SIM reconstruction algorithms that rely on precise estimation of this parameter. On the other hand, using a substantially lower radius (<90% of the pupil) needlessly decreases resolution, especially along the axial dimension. In this work, we sought to position our side beams at 92% of the pupil radius, thereby achieving easily detectable pattern modulation while maintaining high resolution.

After the ratio of the side beam position to pupil radius of BFP *r* and system demagnification factor *M* from the SLM to the sample plane are determined, the corresponding SLM pattern period can be computed as *P* = *Mλ*_*ex*_ / *rNA*, in which *λ*_*ex*_ is the excitation wavelength and *NA* is the numerical aperture of the objective. Taking *λ*_*ex*_ = 488 nm, for example, the pattern period is 45.5 µm for the 1.35 NA silicone oil lens (*M* = 115.7) and 37.6 µm for the 1.27 NA (*M* = 90.1) water lens. Considering the 9.2-µm pixel size of the SLM, these periods correspond to 4.95 pixels and 4.09 pixels, respectively. In practice, the period was fine-tuned for each of the three pattern orientations and different wavelengths, with the goal of achieving *r* = 0.92. The positions of the first-order components of the illumination pattern at the PM are given by *f*_3_*λ*_*ex*_ */* *p*. We machined the PM from a 1-inch-diameter, ∼1-mm-thick aluminum disk, creating seven holes in the disk to selectively transmit only the zero-order and first-order components of the illumination pattern (each hole 0.5–1 mm in diameter, one centered and six spaced at 60° intervals surrounding the central spot). The PM was finely rotated using a mount (Thorlabs, CRM1) to ensure maximal throughput of the illumination pattern.

Third, 3D SIM and four-beam SIM both require the central, zero-order illumination beam to generate the interference pattern at the sample. The relative intensity of the zero-order beam can be controlled by modifying the duty cycle of the SLM pattern, defined by the fraction of pixels in the on-state (π phase retardance) in each period. We set the duty cycle of the SLM pattern to ∼30%, resulting in a zero-order intensity that was 70–75% of the first-order intensity. This ratio was chosen to emphasize the relatively weak amplitudes of the highest lateral spatial frequencies in the illumination pattern, as inspired by previous work^1,3^. In practice, the duty cycle was fine-tuned for each pattern orientation and laser wavelength to maintain the desired intensity ratio (33% and 31% duty cycle for 488-nm and 561-nm lasers, respectively).

With these considerations in mind, to generate SLM patterns with the appropriate orientation, period, duty cycle and relative phase as required for 3D SIM, we adopted the following pattern-finding algorithm (Supplementary Fig. 3). (1) Pick a vector $\overrightarrow A$ parallel to the desired pattern, where the pattern consists of stripes separated by periodicity *p*. $\overrightarrow A$ defines the direction (orientation) of the pattern. (2) Obtain the vector $\overrightarrow B$ perpendicular to $\overrightarrow A$ with length *p*. (3) For any pixel coordinate (*x*, *y*) on the SLM, compute the scalar projection ((*x*, *y*)${\scriptstyle{\cdot\frac{{\overrightarrow{B}}}{{\left\| {\overrightarrow{B}}\right\|}}}}$) of vector (*x*, *y*) onto vector $\overrightarrow B$. (4) Compute the modulo (MATLAB function ‘mod’) after dividing the projection by *p*. If the modulo is greater than the duty cycle multiplied with *p*, a gray level corresponding to 0 phase retardance is assigned to the current pixel. If the modulo is less than the duty cycle multiplied with *p*, a gray level corresponding to π phase retardance is assigned. (5) Repeat the above procedure to find the patterns corresponding to the other phase steps simply by moving the origin of vector (*x*, *y*) along $\overrightarrow B$ by the length of the desired phase step. The advantage of this method is that period and duty cycle may be independently tuned without affecting the pattern orientation. We, thus, found it very useful in fine-tuning the pattern parameters for, for example, multi-color imaging, without needing to change the physical properties of the PM.

Previous 3D SIM work^3,4^ used a binary ferroelectric SLM in which each pixel is set to a binary state, resulting in either 0 or π phase retardance. Here we used a nematic SLM, which allows a greater range of phase values. However, nematic SLMs must be calibrated to generate a look-up table (LUT) that maps the input 0–255 values to the grayscale value that results in a linear 0–2π phase retardance. Before use, we calibrated the SLM for 488-nm and 561-nm wavelengths, following the manufacturer’s guidelines, subsequently selecting the LUT appropriate for the experiment at hand.

To ensure that the illumination pattern has maximum contrast at the sample plane (s-polarization), the LCPR’s state was dynamically adjusted to keep the polarization of illumination beams parallel to vector $\overrightarrow A$ (orthogonal to vector $\overrightarrow B$) for each of the three pattern orientations. Fine alignment of the LCPR optical axis was accomplished using the following procedure. (1) Place the LCPR in a rotation mount (Thorlabs, RSP2D) with one of its input axes roughly aligned with the polarization vector of the illumination post-SLM. (2) Place a linear polarizer after the LCPR with transmission axis in the same direction of vector $\overrightarrow B$. (3) Slowly rotate the LCPR until the transmitted intensity is minimized. Then, fine-tune the control voltage to the LCPR to further reduce the transmitted intensity. (4) Repeat step (3) several times to ensure that the LCPR is properly aligned for the current pattern orientation and repeat step (2) with polarizer axis rotated appropriately to match $\overrightarrow B$ for the other two orientations. (5) Remove the polarizer and record the LCPR input voltages for three pattern orientations and then apply the appropriate voltages to the LCPR during 3D SIM and four-beam SIM experiments (Supplementary Figs. 5 and 6a).

### 3D SIM system alignment

We implemented a series of checks to verify the alignment of our 3D SIM system. First, we minimized aberrations in the wide-field PSF. We used the SLM to produce wide-field illumination by setting all pixels to 0 phase, before acquiring a stack of a single 100-nm bead placed in the center of the FOV. The stack was acquired with near-isotropic voxel size. When projecting the stack to visualize the axial views (*x*–*z* and *y*–*z*), we checked for symmetry of the PSF along each direction. By adjusting the four screws of the Applied Scientific Instrumentation stage insert and the correction collar, we minimized tilt and spherical aberrations, respectively.

Second, we checked the conjugation of the SLM plane to the sample, which is important to achieve imaging with high axial pattern modulation. To optimize this, we acquired a 4-µm 3D SIM stack on a single layer of 100-nm fluorescent beads with axial step size 20–40 nm, which is much smaller than the Nyquist sampling employed when taking biological data. We then used the ‘Illumination Pattern Focus’ calibration tool in SIMcheck^24^ to produce a projection of the axial cross-section of the beads layer along each direction (Supplementary Fig. 12). When the SLM surface is well aligned to the sample plane, axial views of beads at each direction appear symmetric, with approximately equal energy appearing above and below the central intensity maxima. Otherwise, axially projected images appear as a ‘zipper-like’ double layer, which indicates that the detection camera is imperfectly positioned and should be translated axially. For our system, the SLM position and its corresponding axial interference pattern can be optimized only for a single illumination wavelength at once. We prioritized the 488-nm illumination wavelength and corresponding GFP emission band, leaving the camera position unchanged during two-color 3D SIM imaging. However, as indicated below, we did translate the camera for four-beam SIM, two-color acquisition.

Finally, we evaluated the overall performance of the 3D SIM system by imaging 100-nm yellow-green beads and passing the images through SIMcheck (Supplementary Fig. 7). The ‘Raw Fourier Projection’ output was used to check that the first-order illumination pattern components were evident in the frequency domain (Supplementary Fig. 7a). The ‘Motion & Illumination Variation’ output was used to verify that the system remained stable to within the tolerance required for 3D SIM imaging (Supplementary Fig. 7b). A high ‘Modulation Contrast-to-Noise’ value demonstrated good modulation contrast of the illumination pattern (Supplementary Fig. 7c). Statistics from the ‘Reconstructed Intensity Histogram’ indicated a good SNR of the reconstructed image (Supplementary Fig. 7e). Finally, fast Fourier transforms (FFTs) corresponding to the lateral and axial dimensions of the reconstructed image, given by the **‘**Reconstructed Fourier Plots’ (Supplementary Fig. 7f), indicated that imaging performance approached the theoretical resolution limit.

### Mirror for four-beam SIM, four-beam SIM illumination pattern

A half-inch dielectric mirror (Thorlabs, BB05-E02) was glued to a lens tube (Thorlabs, SM05L05) with optical cement (Norland Products), and the entire assembly was mounted to a home-made adaptor. A piezo tip/tilt scanner (Physik Instrumente, S316.10H) connected to the adaptor via set screws was used to precisely control the axial position of the mirror. In this work, only the *z* axis of the piezo scanner was controlled, resulting in pure axial motion. This ‘piezo mirror’ assembly was then mounted on a kinematic mirror mount (Thorlabs, POLARIS-K2T) and a manual translation stage (Newport, 9067) to coarsely adjust the horizontal tilt angle and axial position of the mirror. Finally, the entire setup (Extended Data Fig. 2a) was bolted to the microscope stage and mounted opposite the sample, via half-inch posts, post holders and 90° right-angle clamps (Thorlabs, TR series, PH2 and RA90). By loosening the thumbscrews of the two post holders to remove the assembly, we switched between 3D SIM and four-beam SIM mode. We required an imaging chamber with sufficient clear aperture to accommodate the half-inch diameter of the mirror. This motivated our choice of the magnetic imaging chamber (Warner Instruments, QR-40LP) with 19.7-mm aperture.

In creating the four-beam SIM illumination pattern, we considered two key constraints. (1) The mirror should be close enough (<500 µm) to the coverslip to maintain high coherence between incoming and reflected beams, thereby producing high axial modulation depth. (2) The mirror surface should be perpendicular to the zero-order beam for a purely axial modulation. Fine alignment of the mirror and the reflected beam, necessary to satisfy these constraints, was accomplished using the following protocol. (1) Set the SLM to wide-field mode (all pixels to 0 phase) and focus on a dense single layer of 100-nm beads (or autofluorescence from a dirty coverslip). (2) Attach the mirror setup to the microscope stage with the piezo scanner set at its mid-point voltage. (3) Move the translation stage to lower the mirror toward the sample until it is submerged in the imaging medium. (4) Carefully fine-tune the manual translation stage attached to the mirror (move in ∼10-µm increments) and check the image of the beads layer. When the mirror hits the cover glass, the image of the beads will become out of focus. At this point, move the mirror in the opposite direction (toward the ceiling) by 300 µm. Now the mirror is coarsely positioned in the correct axial position. (5) Acquire a 3D stack of images extending axially over 2 µm, with 20-nm step size. We found that multiple images in the stack provided valuable contextual information, helping us to finely align the system. Manually adjust the knobs on the kinematic mirror mount while monitoring the width and direction of the interference fringes. The closer the mirror is to the correct angle, the wider the fringes become. Perfect two-beam interference with purely axial modulation is achieved when seeing a flat intensity profile (Extended Data Fig. 2c and Supplementary Video 1). (6) Repeat steps (4) and (5) as necessary to ensure that the mirror is properly aligned. Finally, lock the position of the translation stage; at this point, only small adjustments to step (5) are required for same-day alignment when using the same imaging chamber. We note that the ‘side beams’ are also reflected from the mirror but illuminate areas well outside the four-beam imaging field at the illumination angles and mirror-to-coverslip distance used in this work. Although we cleaned the mirror with ethanol between experiments, we found that we never had to replace it during this study.

### Four-beam SIM alignment

A successful SIM reconstruction requires that the axial phase of the illumination (relative position of the illumination pattern with respect to the detection focal plane) is the same for both PSF/OTF measurement and image acquisition. This is usually not a challenge in 3D SIM. However, in four-beam SIM, the axial phase depends not only on the input illumination but also sensitively on the relative position of the mirror with respect to the detection focal plane. The maxima in the axial direction of the interference pattern should ideally coincide with the detection focal plane^20^ (and be maintained at this position), typically requiring the position of piezo scanner (mirror) to be adjusted (and maintained) before commencing four-beam SIM acquisition. We invented a bead-based alignment method to correct phase and drift by using the axial profile of a single 100-nm bead deposited on the coverslip (Supplementary Fig. 9). (1) In wide-field mode, obtain a single image of the sample. It is important that the beads are relatively sparse (for example, 2 µl of methanol solution with 1:20,000 dilution of beads). (2) Select one fiducial bead as an approximation to the PSF, ensuring that there are no other beads within 50 pixels of it. Record its highest intensity coordinate (*x*_0_, *y*_0_). (3) If performing the phase alignment for the first time, acquire a 2-µm stack with 20-nm step size. Otherwise, record a 1-µm stack to reduce photobleaching and improve acquisition speed. (4) Crop the stack’s lateral dimensions to 50 × 50 pixels centered at (*x*_0_, *y*_0_) and apply a 2D Gaussian fit on the maximum intensity projection to estimate the PSF center (*x*_1_, *y*_1_) with subpixel precision. (5) Apply 2D Gaussian fits to each lateral plane in the cropped stack with predefined center (*x*_1_, *y*_1_). Compute the full width at half maximum (FWHM) along the *x* direction *FWHM*(*x*), the *y* direction *FWHM*(*y*) and their average *FWHM*(*average*) at each axial position in the stack. Interpolate *FWHM*(*average*) values within appropriate intervals (for example, 300–500 nm for the yellow-green channel and 400–600 nm for the red channel) with 100 evenly spaced points and then compute the averaged axial positions from the 100 points, yielding ‘PSF offset’. (6) Derive the axial intensity profile of the bead by summing the intensity in each plane over a circular area centered at (*x*_1_, *y*_1_) with a radius of 3 pixels. Fit the axial intensity profile with a 1D Gaussian function to obtain the peak position of the axial interference pattern closest to the ‘PSF offset’—that is, ‘SW peak’. (7) Compute the difference in position between ‘PSF offset’ and ‘SW peak’. If the difference is larger than a set tolerance (for example, 10 nm), the difference is minimized using feedback, by adding ‘PSF offset’ and ‘SW peak’ to the current position of the piezo stage (moving the sample) and piezo scanner (moving the mirror), respectively. Steps (3) to (7) are repeated until the desired tolerance is achieved. Once the difference between ‘PSF offset’ and ‘SW peak’ is within the set tolerance, the axial intensity profile should exhibit side lobes of equal height above and below focus (Supplementary Fig. 8b), and four-beam SIM imaging can commence using the current piezo scanner and piezo stage settings. After moving the stage to image another FOV, we usually waited ∼5 minutes before commencing imaging, finding that this settling time helped to stabilize the system. For all the single-timepoint imaging in this work, we applied this bead-based alignment protocol only once, before performing four-beam SIM acquisition. If time-lapse four-beam SIM imaging is required, the frequency of fiducial-based correction may be adjusted as desired.

As discussed above, the axial interference pattern of 3D SIM can be optimized only for a single illumination wavelength. If the camera position (detector plane) is adjusted correctly for the 488-nm illumination, the corresponding order 1 OTF fully overlaps in four-beam SIM, which is sufficient for reconstruction (Supplementary Fig. 12a). However, when imaging red fluorescent beads excited with 561-nm illumination, gaps appear in the order 1 OTF if the detector plane is not moved (Supplementary Fig. 12b). Such missing frequency components cannot be restored during Wiener reconstruction. After translating the camera to the optimal position for 561-nm illumination, OTF overlap is restored (Supplementary Fig. 12c). Thus, to achieve acceptable two-color four-beam SIM reconstructions, we recorded the optimal positions for 488-nm and 561-nm illumination wavelengths and switched the camera positions between color acquisitions. We found that the minimum distance the camera needs to move between each color acquisition is 1.74 mm. Over this distance, we could discern no obvious spherical aberration when imaging red beads with 561-nm illumination. Moving the translation stage seating the camera takes ∼10 seconds between colors. We note that this temporal offset can be eliminated by adding a second emission path—that is, a dichroic mirror and another camera whose axial position is optimized specifically for 561 nm.

### 3D SIM and four-beam SIM data acquisition and OTF generation

Raw 3D SIM and four-beam SIM data were collected by applying patterned illumination with 5 phases (2π/5 relative spacing) and 3 orientations (60° apart) for a total of 15 images per plane before changing focus (125-nm step size for 3D SIM and 60-nm step size for four-beam SIM). Each image stack was, thus, saved in XYPAZ format, where ‘XY’, ‘P’, ‘A’ and ‘Z’ denote lateral plane, phase, orientation and depth, respectively.

PSF data were acquired similarly except that only one pattern orientation (the first orientation) was acquired when imaging a single 100-nm fluorescent bead. For four-beam SIM PSF measurement, phase alignment and drift correction were also applied immediately before acquisition. The axial range of the acquired PSF was 8 µm (4 µm below and above the bead center), and the image was cropped to a 256 × 256-pixel region centered on the bead. The OTFs required for parameter estimation and Wiener reconstruction were derived using the following procedure. (1) Sum all five image volumes (corresponding to the five phases) to obtain an estimate of the wide-field PSF. Use three-point parabolic fitting around the pixel with maximum intensity to estimate the lateral center of the PSF more precisely. To estimate the axial center of the PSF, apply another three-point (3D SIM) or five-point (four-beam SIM) parabolic fitting on the axial view. (2) Subtract camera background, soften edges by multiplying each of the five image volumes with a squared sine function and convert them to the frequency domain by computing the 3D FFT. Multiply the FFT results with a separation matrix to obtain the real and imaginary parts of the five information bands. Multiply the bands with a phase factor determined by the PSF center to shift the PSF to the origin. (3) Divide the bands with the Fourier transform of a solid sphere with a diameter of the bead (100 nm) to compensate for the finite bead size. (4) Rotationally average along *k*_*z*_ axis to reduce noise, converting the bands into 2D data (*k*_*r*_/*k*_*z*_ view). (5) The OTF support of the wide-field microscope is a toroid with extent determined by the detection NA and emission wavelength. All values outside the OTF support were set to 0 to eliminate noise. In 3D SIM, order 0 and 2 OTFs are identical to the wide-field OTF, but the order 1 OTF is composed of two overlapping wide-field OTFs. In four-beam SIM, only the order 2 OTF is equivalent to the wide-field OTF, as the order 0 OTF consists of three separated wide-field OTFs, and the order 1 OTF consists of four overlapping wide-field OTFs (Supplementary Fig. 12). (6) Normalize all OTFs to the maximum value (DC coordinate) of the order 0 OTF, setting this value to 1. OTFs must be measured for each objective and each laser wavelength and can be subsequently applied to reconstruct SIM data acquired under the same conditions. When performing SIM reconstruction (see the following section), the 2D OTFs must be converted back into a 3D form. We established this correspondence by assigning each voxel coordinate (*k*_*x*_, *k*_*y*_, *k*_*z*_) in the desired 3D OTF to a 2D coordinate (*k*_*r*_, *k*_*z*_) = $\left( {\sqrt {k_x^2 + k_y^2} ,k_z} \right)$, obtaining the value at each 2D coordinate by interpolating the values from the four nearest pixels.

### 3D SIM and four-beam SIM image reconstruction

Raw 3D SIM and four-beam SIM data were processed with custom-developed MATLAB (MathWorks, R2021b) software. We note that four-beam SIM reconstruction is fundamentally no different than 3D SIM. Although additional axial information is present in four-beam SIM data, such data contain the same number of lateral illumination frequency components and, thus, lateral information bands as in 3D SIM. The reconstruction algorithm we use is, thus, based on traditional 3D SIM reconstruction^1^ and can be subdivided as follows. (1) Pre-processing. A constant camera background was first subtracted from the raw data, and the resulting image edges were softened by multiplying the images with a squared sine function. All images were normalized to have the same total intensity to compensate for fluctuations in illumination or bleaching between the different illumination phases, illumination orientations or sample depths. (2) Frequency unmixing. For each orientation, five information bands (corresponding to m = 0, ±1 and ±2 lateral illumination frequency components) can be estimated and separated based on the 3D FFTs of the five input volumes. (3) Parameter estimation. The precise position of the frequency components (corresponding to pattern line spacing and angle), the initial phase and the corresponding modulation depth of order 2 (the highest order) were determined by computing the cross-correlation between the shifted order 2 and order 0 (DC) bands. The modulation depth is a useful indicator of cross-correlation performance as line spacing and angle are finely varied to find their optimal values. Good parameter estimation occurs when the modulation depth reaches a local maximum, maximizing the overlap between order 0 and the shifted order 2 information bands. Half the value of the order 2 illumination frequency component position was used for positioning order 1. The estimated illumination frequency component of order 1 was then used to determine the initial phase and modulation depth of order 1. (4) Wiener reconstruction. A generalized Wiener filter incorporating all estimated parameters shifted the bands to their correct positions in frequency space and combined all three orientations to fill in the ‘missing cone’ and enhance resolution. Using a Wiener parameter that is too high reduces resolution, whereas using a Wiener parameter that is too low artificially increases noise. A value of 0.001, used throughout this work, was empirically found to give a good balance between resolution and noise. After multiplying the generalized Wiener filter result with a triangular apodization function to suppress high-frequency edge artifacts, the final SIM images were derived by computing the inverse FFT and keeping non-negative real values.

### Hardware control

A PC computer (@Xi workstation, Intel Xeon CPU E5-1660 v4 @ 3.20 GHz, 16 threads, 64 GB memory and 1 TB M.2 NVME SSD) was used to issue commands (waveforms) to a NI-DAQ card (National Instruments, PXI 6733, BNC 2110) housed in an external data acquisition (DAQ) card chassis (National Instruments, PXIe-1073), the internal pco.edge camera acquisition card and the internal SLM control card (Supplementary Fig. 4). A schematic describing the main waveforms (and their relative timing) is shown in Supplementary Fig. 5. The camera was set to external trigger with rolling shutter mode, receiving rising edges from a digital output (DO) on the DAQ card. The SLM was also set to external trigger mode, receiving falling edges from another DO port to change patterns in a predefined sequence. Stepwise waveforms from an analog output (AO) were used to drive the piezo stage for volumetric acquisition. Three AO ports (488 nm, 561 nm and blanking) drove the AOTF to individually control the intensity of each illumination wavelength. Three different voltages from an AO port were supplied to the LCPR to maximize modulation depth for the three pattern orientations. In four-beam SIM, the piezo mirror position was controlled by an additional AO port during phase alignment and remained unchanged during subsequent four-beam SIM acquisition. The *x* and *y* positions of the Applied Scientific Instrumentation stage, the *z* position of the Applied Scientific Instrumentation objective stepper motor (for coarse axial objective positioning) and the filter wheel selection were controlled through serial port commands.

Control software (Python 3.7.1) was based on our previous iSIM control software^60^ but modified substantially to control the SLM, LCPR and piezo scanner necessary for 3D SIM and four-beam SIM. Timing diagrams (Supplementary Figs. 5 and 6) show key use cases, including PSF/OTF data acquisition (Supplementary Fig. 5a), 3D SIM and four-beam SIM acquisition at one lateral plane (Supplementary Fig. 5b), volumetric acquisition (Supplementary Fig. 6a) and axial alignment between illumination pattern and the detection focal plane (Supplementary Fig. 6b).

Volumetric acquisition time was determined by the laser exposure time per image, number of rows of pixels in the image, camera readout time (delay time between external trigger and laser exposure), SLM loading time, LCPR switching time and the number of z slices. The laser exposure time was set to 20 ms (50 ms in Fig. 4e–k and Supplementary Fig. 15); the raw image size was 1,280 × 1,080 pixels; the camera readout time was 5.27 ms; the SLM loading time was 5 ms; and the LCPR switching time was 10 ms. Thus, ∼25 ms is required per phase (single image), ∼130 ms for one orientation (five images) and ∼390 ms for one plane (3 orientations × 5 phases = 15 images). For example, in the fixed U2OS cell imaging shown in Fig. 2e–g, when setting the z step size at 0.125 µm for 3D SIM and 0.06-µm z step size for four-beam SIM, we collected a volume spanning 90 µm × 76 µm × 4 µm in ∼12.7 seconds and ∼26.5 seconds, respectively. We also employed a technique based on the ‘Transient Nematic Effect’ to shorten the temporal response of the LCPR by ∼2-fold. When changing the LCPR polarization state from low to high voltage, a very short duration (2 ms) high-voltage spike is used to accelerate the molecular alignment parallel to the applied field. Voltage is then reduced to achieve the desired polarization (Supplementary Fig. 5b) in the remaining 8 ms of the 10-ms total switching time. Similarly, when changing from high to low voltage, a zero-voltage spike (2 ms) is applied before targeting the desired voltage (Supplementary Fig. 6a). For two-color imaging with only one timepoint, 561 nm always preceded 488 nm to reduce photobleaching. Time-lapse imaging consisted of a series of repetitions of single-volume imaging with or without a delay between timepoints. Supplementary Table 5 details the volume size and corresponding acquisition time for all the data presented in the paper.

### Registration and bleach correction of volumetric time-lapse data

The output values after deep learning are floating-point numbers between 0 and 1. To guarantee that all volumes have similar background intensity values for supplementary videos, outputs were converted to 16-bit unsigned integers and then bleach-corrected using the ImageJ plugin Bleach Correction (Image → Adjust → Bleach Correction → Histogram Matching). This plugin is available at https://imagej.net/plugins/bleach-correction. Adjacent timepoints were registered using a GPU-based 3D affine registration method^49^, available at https://github.com/eguomin/regDeconProject/tree/master/RegistrationFusion. This registration method is written in C++/CUDA to generate a DLL file, which is then called in MATLAB. The ‘translation only’ registration mode was used.

### Segmentation and tracking in live microtubule data

To track filaments, raw image data were first manually segmented in Aivia 10.2 (Leica Microsystems) to create mask channels. Mask channels were incorporated as a separate channel in Imaris 9.8.2 (Bitplane) and then converted to surface objects and overlaid over the input data. Microtubule buckling and MTOC movements were manually tracked in Imaris by placing spots at the desired locations, with the position of the MTOC identified as the point of convergence of microtubule filaments.

### Simulation of mixed structures for axial resolution enhancement

We simulated 3D images (50 pairs for training and ten for testing) with mixed structures of dots, lines and hollow spheres for validating the six-direction deep learning method that yields isotropic resolution from raw 3D SIM input (Supplementary Fig. 13a). Simulated structures were created in MATLAB (MathWorks, R2019b, with the Imaging Processing Toolbox). Each volume was composed of 3,000 dots, 1,800 lines and 600 hollow spheres, randomly located in a 300 × 300 × 300 grid (assuming a pixel size of 40 nm). Dots were generated with random intensity (2,000–7,000 counts); lines were generated with random angles in 3D (1–360° relative to axial axis and 1–360° in lateral planes), random lengths (1–72 pixels) and a random intensity (100–500 counts); and hollow spheres were generated with random inner diameter (2–20 pixels), random thickness (1–2 pixels) and random intensity (10–200 counts). Structures were blurred with different 3D Gaussian functions. In the first dataset, sigma values were 1.3, 1.3 and 3.7 pixels in *x*, *y* and *z* dimensions, respectively, which represented the raw 3D SIM volumes with a resolution of 125 × 125 × 350 nm^3^ (FWHM value), assuming a pixel size of 40 nm. In the second set, sigma values were all 1.3 pixels, serving as the ground truth dataset with an isotropic resolution of 125 × 125 × 125 nm^3^ for the calculation of SSIM and PSNR.

For the six-direction deep learning method, we generated two sets of volumes based on the first dataset (that is, the simulated raw 3D SIM input), one blurred with a 2D Gaussian function at each *x*–*z* slice (sigma = 3.6 pixels in *x* dimension and 0.5 pixels in *z* dimension), serving as the ‘input’ in the training; the other blurred with a 1D Gaussian function (sigma = 0.5 pixels) along the *z* dimension, serving as the ‘ground truth’ in the training. To study the effects of axial downsampling factors (Supplementary Fig. 13b), we extracted *x*–*y* planes from the first dataset with an axial interval of 2, 4, 6 and 8 (that is, downsampling factor) to create new volumes with 300 × 300 × 150 voxels, 300 × 300 × 75 voxels, 300 × 300 × 50 voxels and 300 × 300 × 37 voxels, respectively. For each downsampling factor, we interpolated the volumes back to 300 × 300 × 300 voxels and repeated the same Gaussian blurring process as for the first dataset (that is, without any downsampling) to generate ‘input’ and ‘ground truth’ datasets in the training.

For the prior isotropization method^35^, we created a 2D PSF by Gaussian blurring a dot along the *y* dimension (sigma = 3.6 pixels) and fed this into the content aware image restoration (CARE^35^) network pipeline, blurring the lateral views to resemble the lower resolution axial views and learning to reverse this blur to achieve isotropic resolution.

### Quantitative analysis

For the simulated datasets (Supplementary Fig. 13a), we selected 10 volumes to evaluate the structural similarity index measure (SSIM) and peak signal-to-noise ratio (PSNR) of the results that we obtained using our six-direction deep learning method versus the ground truth with MATLAB (MathWorks, R2019b), and then we computed the mean value and standard deviation of these volumes (Supplementary Fig. 13b).

Lateral and axial resolution measures derived from fluorescent beads (Fig. 1g and Extended Data Fig. 3d) were estimated by computing the FWHM of line intensity profiles along *x*–*y* and *x*–*z* views. Statistical results (mean ± s.d.) were obtained from *n* = 102, 100 and 99 beads (1.35 NA) and 85, 81 and 78 beads (1.27 NA) for wide-field microscopy, 3D SIM and four-beam SIM, respectively. For rough resolution comparisons, we plotted the Fourier transforms of the images (Fig. 1e, insets, and Extended Data Fig. 3b, insets).

### Neural networks for denoising and axial resolution improvement

We adapted and developed five neural networks including (1) CARE^35^ network that provides isotropic output (that is, prior isotropic method); (2) six-direction deep learning method that improves axial resolution, which was implemented with the CARE network; (3) two-step 3D RCAN^37^—that is, first denoising raw, low SNR 3D SIM volumes and then predicting 3D SIM from the Wiener reconstruction of the denoised 3D SIM volumes (Fig. 5a and Supplementary Fig. 17a); (4) 15-input 3D RCAN that can directly predict a 3D SIM reconstruction from 15 raw low SNR 3D SIM volumes (Supplementary Fig. 18b); and (5) 15-input DenseDeconNet for predicting 3D SIM reconstructions from 15 low SNR 3D SIM volumes (Supplementary Fig. 18b). Most model training and applications were performed on an NVIDIA TITAN RTX GPU (with 24 GB memory) installed on a local workstation. We also performed some CARE and RCAN training and model applications with Amazon Web Services, using a virtual machine with four NVIDIA Tesla V100 GPUs (each with 16 GB memory). We used Python version 3.7.1 for all neural networks and TensorFlow framework versions 2.4.1, 1.13.1 and 1.14.0 for CARE, RCAN and DenseDeconNet, respectively.

CARE software was installed from GitHub (https://github.com/CSBDeep/CSBDeep). For simulated data training (Supplementary Fig. 13a), patches of size 64 × 64 × 64 voxels were randomly cropped from 50 3D volumes with size 300 × 300 × 300 voxels. For other 3D datasets (Figs. 4–6, Extended Data Fig. 4 and Supplementary Figs. 13–20), 3D SIM volumes were first interpolated to 50-nm pixel size in all three dimensions. When using the prior method (Supplementary Fig. 13), the interpolated volumes, a 2D PSF (consisting of a point blurred with a 1D Gaussian function, sigma = 2.8 pixels along the *y* dimension) and the axial downsampling factor were fed into the isotropic CARE network using a patch size of 64 × 64 to create training pairs.

For the six-direction deep learning method, interpolated volumes were (1) blurred with a 1D Gaussian function (sigma = 1.0 pixel) along the *z* dimension to remove spurious sidelobes in the Fourier domain due to axial interpolation, serving as the ‘ground truth’ in the training or (2) blurred with a 2D Gaussian function at each *x*–*z* plane (sigma = 2.8 pixels in the *x* dimension, degrading lateral resolution to the extent of the axial resolution, and 1.0 pixel in the *z* dimension as for the ground truth); downsampled along the *x* dimension to mimic the coarse axial sampling in real experiments; and, finally, upsampled to recover an isotropic pixel size, serving as the ‘input’ for training (Supplementary Fig. 14a). Both downsampling and upsampling use bilinear interpolation. Patches of size 64 × 64 × nz (nz is the number of planes) were randomly cropped from the data, and 10% of the patches were set aside for validation.

The trained network can recover resolution along the *x* axis from unseen ‘test’ data (Supplementary Fig. 14b), which were degraded with the same procedure (that is, blurring, downsampling and upsampling) as the ‘input’ data used to train the network. Digitally rotating the degraded data about the *y* axis and passing the data through the trained network results in improved resolution along the lateral direction in the rotated space. By rotating the data back to the original frame, resolution can, thus, be improved along an arbitrary axis (Supplementary Fig. 14c). Finally, by recording the maximum value at each spatial frequency taken over all rotations, a final prediction with isotropic resolution is obtained (Supplementary Fig. 14d).

Naive rotation and interpolation in the *x*–*z* plane will markedly enlarge the size of each volume. For example, for raw input data with large *x*–*z* aspect ratio spanning 800 × 800 × 80 voxels, as is typical for imaging single cells, a 60° rotation in the *x*–*z* plane will produce a volume 800 × 800 × 733 voxels in size. This large size results in inefficient network prediction (for this example, the prediction is useless over a region spanning 800 × 800 × 653 voxels). We, thus, cropped the input data into multiple subvolumes (for example, 10 subvolumes of 80 × 800 × 80 voxels) before application of the CARE network. After rotation and interpolation, each subvolume spanned only 110 × 800 × 110 voxels, resulting in useless prediction over only 30 × 800 × 30 voxels, a 65-fold improvement in efficiency compared to the non-cropped case. This processing pipeline was implemented in Python version 3.7.1: (1) automatically cropping raw input data to multiple subvolumes with identical *x* and *z* dimensions, with 10-pixel overlap along the *x* axis; (2) rotating and bilinearly interpolating each subvolume with the built-in SciPy function ndimage.rotate; (3) applying the neural network model to each subvolume; (4) rotating back the subvolume predictions and cropping them to their original sizes; (5) stitching the subvolumes by averaging the overlap regions; (6) Fourier transforming the stitched volume for each rotation angle; (7) calculating the maximum value at each spatial frequency taken over all rotations; and (8) inverse Fourier transforming the result and taking the absolute values as the final output.

All training for the prior method and the six-direction method used 50 volumes without data augmentation (rotation and translation), and testing varied from 10 to 100 volumes (Figs. 4–6, Extended Data Fig. 4 and Supplementary Figs. 13, 15–17 and 20). The training learning rate was 2 × 10^4^; the number of epochs was 100; the number of steps per epoch was 200; and the mean absolute error (MAE) was used as loss function. The training time for each model varied from ∼3 hours to 7 hours. For example, it took ∼3 hours for training the Jurkat T cells expressing EMTB 3× GFP datasets (Fig. 6 and Supplementary Videos. 13–16) and ∼5.5 hours to apply the model to recover a 100-timepoint dataset with size 420 × 420 × 80 voxels and six directions (total 600 volumes), including the processing time for cropping, image rotation, network prediction, rotation back, stitching, combination with maximum frequency method and file reading/writing.

For the two-step denoising studies employing RCAN (Figs. 5 and 6 and Supplementary Figs. 17–20), we used our recently developed 3D RCAN model, appropriate for restoring image volumes (https://github.com/AiviaCommunity/3D-RCAN)^37^. For all 3D data, training patches of size 128 × 128 × nz (nz is the number of acquisition planes without axial interpolation) were randomly cropped from the data. To train the first denoising network, we used patches derived from 750 matched volumes (50 acquisitions × 3 orientations × 5 phases) acquired at low and high illumination intensity (for example, ∼0.8 W/cm^2^ versus 8 W/cm^2^ for Supplementary Fig. 17). The trained network was used to denoise raw, low SNR data (5 phases × 3 orientations) and the denoised output used for 3D SIM reconstructions as described above. To train the second denoising network, we used patches derived from 50 matched volumes (3D SIM reconstructions derived from high SNR raw data as high SNR ‘ground truth’ and the 3D SIM reconstructions derived from the denoised data as the matched ‘input’ data). The output of this second model may then be axially interpolated, blurred, downsampled, upsampled and fed into the six-direction model that enhances axial resolution, generating a final denoised reconstruction with isotropic resolution (Figs. 5 and 6 and Supplementary Figs. 17 and 20). For training the RCAN networks, the learning rate was 2 × 10^−4^; the number of epochs for training was 200; the number of residual blocks was five; the number of residual groups was five; the number of channels was 32; the number of steps per epoch was 400; and the MAE was used as the loss function.

For some studies (Supplementary Figs. 18 and 19), we extended our 3D RCAN method to handle 15 input volumes. Multiple volumetric inputs with the same shape were concatenated into a single volume with multiple channels and fed to the model. We followed ref. ^62^ and used subpixel convolution^63^ to generate upsampled super-resolution outputs. A fixed number of volumetric inputs is paired with one ground truth volume for model training, and the same number of volumetric inputs is expected for prediction. For this multiple input training, we used the same network parameters as in single-input RCAN training.

For the studies using DenseDeconNet (Supplementary Fig. 18), we similarly extended our previously published single-input neural network (https://github.com/eguomin/regDeconProject/tree/master/DeepLearning) for 15 inputs. The input data were 15 raw low SNR 3D SIM volumes, and the ground truth data consisted of high SNR 3D SIM reconstructions. For all 3D data, patches of size 64 × 64 × 64 were randomly cropped from the data. All 15 low SNR data were concatenated along the channel axis to form the input. The percentile-based normalization was adopted to normalize the input and ground truth (the low percentile was 1.0, and the high percentile was 99.8). In the training, the objective function incorporated three terms: the mean square error (MSE), the structural similarity (SSIM) index and the minimum value of the output (MIN); the parameter to control the MIN term was set to 1. The training epoch was 200 with 400 steps per epoch; the learning rate was 0.01; the decay rate was 0.985; and the decay step was 400.

### Sample preparation

#### General considerations

Coverslips were cleaned and coated with poly-l-lysine and (particularly for four-beam SIM) fluorescent beads. Unless otherwise noted, we used the following protocol. High-precision #1.5 coverslips (Thorlabs, CG15XH) were cleaned by immersion in 75% ethanol overnight and air dried before use. Approximately 50 µl of poly-l-lysine solution (Sigma-Aldrich, P8920) was applied to the center of the coverslips in a biosafety cabinet. After air drying for 15 minutes at room temperature, coverslips were rinsed in pure ethanol and air dried until use. Orange, red or yellow-green FluoSpheres (Invitrogen, F8800, F8801 and F8803, all 0.1-µm diameter) were selected depending on the application and dissolved in pure methanol at 1:20,000 dilution, and 2 µl of the solution was applied to the center of a poly-l-lysine-coated coverslip.

For four-beam SIM experiments using the 1.35 NA silicone oil lens, we performed imaging in an iodixanol solution index-matched to the RI of the silicone oil (1.406) to minimize aberrations. Iodixanol solution (Sigma-Aldrich, D1556) consisted of 45.6% iodixanol in water. We verified its RI as 1.406 using a refractometer (American Optical).

#### Bacteria

Vegetatively growing *B. subtilis* strain PY79 (ref. ^64^) was grown in LB broth (KD Medical, BLE-3030) for 2 hours at 37 °C, shaking at 250 r.p.m. To visualize localization of DivIVA-GFP, strain KR541 (*amyE::P*_*hyperspank*_*-divIVA-gfp cat*)^65^ was grown in casein hydrolysate media^66^ (KD Medical, CUS-0803) containing 1 mM final concentration of IPTG to induce DivIVA-GFP production for 2 hours at 37 °C, shaking at 250 r.p.m. To induce sporulation by the resuspension method, an overnight culture of strain CVO1000 (*amyE::spoVM-gfp cat*)^67^ grown at 22 °C was first subcultured to a final OD_600nm_ of 0.1 in casein hydrolysate media for 2 hours at 37 °C, shaking at 250 r.p.m. Cells were harvested by centrifugation at 14,000*g*, resuspended in an equal volume of Sterlini–Mandelstam media^66^ (KD Medical, CUS-0822) and grown for 4 hours at 37 °C, shaking at 250 r.p.m. Before imaging, 1 ml of culture was removed and centrifuged at 14,000*g*, and the cell pellet was resuspended in PBS.

For some experiments, we stained bacterial membranes. Bacteria were diluted in 1 ml of PBS and stained with CellBrite Fix 488 (Biotium, 30090-T) or CellBrite Fix 555 (Biotium, 30088-T) for 5 minutes at room temperature at 1× working concentration according to the manufacturer’s guidelines. Stained bacteria were washed three times with 1× PBS, each time centrifuging the solution at 3,000 r.p.m. for 3 minutes. Finally, bacteria were concentrated in 100 µl of 1× PBS, and 2 µl was placed on the center of a poly-l-lysine and beaded coated coverslip for imaging.

#### Mouse LSECs

C57BL/6 mouse primary LSECs (Cell Biologics, C57-6017) were cultured in culture medium (Complete Mouse Endothelial Cell Medium, containing 10% FBS and Endothelial Cell Growth Supplement (Cell Biologics, M1168, 6912 and 1166, respectively)) according to the manufacturer’s instructions. Before cell deposition, glass coverslips (Thorlabs, CG15XH) were sequentially washed with 0.1 M sodium hydroxide, 0.1 M hydrochloric acid and acetone, coated with 200 μl of poly-l-lysine solution (Sigma-Aldrich, P8920), allowed to dry for 5 minutes and washed once with pure methanol. Equal volumes of red (Invitrogen, F8801) and yellow-green (Invitrogen, F8803) fluorescent microspheres were diluted 1:20,000 in methanol, and 2 μl of the bead dilution was added to the center of the coverslip. After the methanol dried, the coverslips were placed in an aqueous solution containing 1% rat tail collagen (Cell Biologics, 6953) for 1 hour and placed in culture medium. After counting, 50,000 cells were seeded per 3.5-cm dish and incubated at 37 °C in 5% CO_2_ for 16 hours. Cultured cells were fixed with 4% paraformaldehyde in PBS for 20 minutes, washed in PBS, incubated with Alexa Fluor 568 phalloidin (Thermo Fisher Scientific, A12380, 7 U ml^−1^) in PBS for 2 hours at room temperature, washed in PBS, fixed again in 4% paraformaldehyde, washed with water, stained with CellBrite Fix 488 (Biotium, 30090-T, 1:250 dilution) in PBS for 10 minutes at room temperature and, finally, washed and stored in PBS before imaging. Imaging was performed within 6 hours of staining.

#### MEFs

MEFs (gift of Oliver Daumke’s laboratory) were cultivated in DMEM (Gibco, 119950409) supplemented with 10% FBS (Atlanta Biologicals, S10350) and 1% penicillin–streptomycin (Gibco, 15070063) at 37 °C in 5% CO_2_. For imaging experiments, the cells were seeded on fibronectin (Sigma-Aldrich, F1141) coated glass coverslips (Thorlabs, CG15XH) and transfected with caveolin1-EGFP plasmid (gift of Richard Lundmark) using Lipofectamine 3000 (Invitrogen, L3000-001). After 48 hours, the cells were washed with PBS (Gibco, 10010023), followed by fixation with 4% paraformaldehyde/PBS (Electron Microscopy Sciences, 15700) for 20 minutes. Next, the cells were washed three times with PBS, treated with 3% BSA (Sigma-Aldrich, A9418)/0.1% Triton X-100 (Sigma-Aldrich, X100)/PBS for 30 minutes, followed by incubation with 3% BSA/PBS for 45 minutes. Caveolae were stained with rabbit polyclonal antibody against cavin1 (Abcam, 76919, 1:100 in 3% BSA/PBS) for 1 hour. Afterwards, MEFs were washed extensively, and GFP-booster (tagged with Alexa Fluor 488, ChromoTek, gb2AF488-50, 1:500 in 3%BSA/PBS) and anti-rabbit Alexa Fluor 568 (Invitrogen, A11011, 1:500) were applied for 1 hour. To remove any unbound antibodies, cells were washed five times with PBS and stored at 4 °C in PBS until imaging.

#### Jurkat T cells

E6-1 Jurkat cells were cultured in RPMI 1640 supplemented with 10% FBS and 1% penicillin–streptomycin antibiotics. For transient transfections, we used the Neon (Thermo Fisher Scientific) electroporation system 2 days before the experiment. In total, 2 × 10^5^ cells were resuspended in 10 μl of R-buffer with 0.5–2 μg of the EMTB 3× EGFP plasmid (a gift from William Bennett, Addgene plasmid 26741). Cells were exposed to three pulses of amplitude 1,325 V and duration 10 ms in the electroporator. Cells were then transferred to 500 μl of RPMI 1640 supplemented with 10% FBS and kept in the incubator at 37 °C.

Glass coverslips (Thorlabs, CG15XH) were incubated in poly-l-lysine (Sigma Aldrich, P8920-100ML) at 0.1% w/v for 10 minutes. Poly-l-lysine was washed with 70% ethanol, and the coverslips were left to dry. T-cell-activating antibody coating was performed by incubating the coverslips in a 10 μg ml^−1^ solution of anti-CD3 antibody (Hit-3a, Thermo Fisher Scientific, 16-0039-85, 1:100 dilution in 1× PBS) for 2 hours at 37 °C. Excess anti-CD3 was removed by washing with L-15 imaging media (Thermo Fisher Scientific, 21-083-027) immediately before cell plating.

For fixed cell experiments, EMTB 3× EGFP-expressing Jurkat cells were plated on anti-CD3-coated coverslips for 7 minutes in L-15 media. Cells were rinsed with 1× PBS three times at room temperature, covered with 100% methanol cooled to −20 C for 3 minutes and, finally, washed three times with 1× PBS.

For experiments with live cells, a small volume (typically 500 µl) containing 3 × 10^5^ cells were centrifuged for 5 minutes at 250*g*. The supernatant was removed; the cells were resuspended in 120 µl of L-15 imaging media; and approximately 40 µl (equivalently 1 × 10^5^ cells) of L-15 media with cells was added to the anti-CD3-coated coverslip. Cells were allowed to settle for 2 minutes before commencing imaging.

#### U2OS cells

U2OS cells (American Type Culture Collection, HTB-96) were cultured in DMEM media (Lonza, 12-604F) supplemented with 10% FBS (Thermo Fisher Scientific, A4766801) at 37 °C and 5% CO_2_ on poly-l-lysine and optionally beaded (for four-beam SIM) coverslips placed in six-well plates (Corning, 3506).

We used the following buffers for immunolabeling vimentin and microtubules. (1) PEM buffer (in 1× PBS): 80 mM PIPES sodium salt (Sigma-Aldrich, P2949), 5 mM EGTA (Sigma-Aldrich, E3889) and 2 mM MgCl_2_ (Quality Biological, 351-033-721), adjusted with sodium hydroxide to pH 6.8. (2) Pre-extraction solution: PEM buffer supplemented with 0.3% Triton X-100 (Sigma-Aldrich, 93443) and 0.125% glutaraldehyde (Sigma-Aldrich, G5882). (3) Microtubule fixation buffer: pre-extraction solution supplemented with 2% paraformaldehyde (Electron Microscopy Sciences, 15710).

To immunolabel microtubules, U2OS cells were treated with pre-extraction buffer at 37 °C for 30 seconds, and then the buffer was quickly replaced with microtubule fixation buffer at 37 °C for 15 minutes. Fixed cells were rinsed three times with 1 ml of 1× PBS (Gibco, 10010-023), quenched in 1 ml of 0.1% sodium borohydride/PBS solution (Sigma-Aldrich, 213462) for 7 minutes at room temperature and blocked in 100% FBS (Sigma-Aldrich, 12103 C) at 37 °C for 1 hour. Microtubules were incubated with primary mouse-α-tubulin antibody (Invitrogen, 32-2500, 1:100) in 1× PBS supplemented with 10% FBS overnight at 4 °C, washed three times (1-minute incubation each time) in 1× PBS and labeled with secondary donkey-α-mouse Alexa Fluor 488 antibody (Jackson ImmunoResearch, 715-547-003, 1:200 dilution) in 1× PBS supplemented with 10% FBS for 1 hour at room temperature.

Vimentin was immunolabeled with primary rabbit-α-vimentin (Abcam, 92547, 1:100) in 1× PBS supplemented with 10% FBS overnight at 4 °C, washed three times (1-minute incubation each time) and labeled with secondary donkey-α-mouse Alexa Fluor 594 (Jackson ImmunoResearch, 711-587-003, 1:200 dilution) in 1× PBS supplemented with 10% FBS for 1 hour at room temperature. Samples were rinsed three times in 1× PBS before imaging. For dual-color samples, we performed microtubule and vimentin immunolabeling in parallel on the same sample.

For immunolabeling Tomm20, U2OS cells were fixed with 2% paraformaldehyde and 0.125% glutaraldehyde in 1× PBS for 15 minutes at room temperature. Cells were rinsed three times with 1× PBS, permeabilized by 0.1% Triton X-100/PBS (Sigma-Aldrich, 93443) for 1 minute at room temperature, rinsed three times with 1× PBS, incubated with primary rabbit-α-Tomm20 (Abcam, ab186735, 1:100 dilution in 1× PBS) for 1 hour at room temperature, washed in 1× PBS (1-minute incubation each time) three times, stained with secondary antibody donkey-α-rabbit Alexa Fluor 488 (Jackson ImmunoResearch, 711-547-003, 1:200 in 1× PBS) for 1 hour at room temperature and, finally, washed three times (1-minute incubation each time) before imaging.

To label actin, U2OS cells were similarly fixed, permeabilized and washed and then incubated with phalloidin Alexa Fluor 488 (Invitrogen, 2090563, 1:50 dilution in 1× PBS) or phalloidin Alexa Fluor 568 (Invitrogen, 1800130, 1:50 dilution) for 1 hour at room temperature, rinsed three times with 1× PBS and incubated in 1× PBS or iodixanol solution before imaging.

We stained mitochondria and internal membranes with synthetic dyes for live cell imaging. In the former case, U2OS cells were incubated with MitoTracker Green FM (Invitrogen, M7514, 100 nM in 1× PBS) for 15 minutes at 37 °C and rinsed three times with 1× PBS immediately before imaging. In the latter case, U2OS cells were incubated in DMEM media with Potomac Gold (kindly provided by Luke Lavis; 200 nM in DMEM media supplemented with 10% FBS) for 30 minutes at 37 °C. Immediately before imaging, cells were rinsed three times with 1× PBS.

We also transfected cells with plasmids to mark organelles for live imaging experiments. Cell cultures were transfected using X-tremeGENE HP DNA Transfection Reagent (Sigma-Aldrich, 6366236001). The transfection mixture contained 100 μl of 1× PBS, 2 μl of transfection reagent and 1 μg of plasmid DNA. To label outer mitochondrial membranes, cell cultures were transfected at 50% confluency with mEmerald-Tomm20-C-10 plasmid DNA (Addgene, 54281) and imaged 1 day after transfection. We also created a stable cell line to mark lysosomes. Cells were transfected with LAMP1-EGFP plasmid DNA (gift of George Patterson’s laboratorry) at 80–85% confluency. The next day, cells were incubated with fresh media for another 24 hours. Transfected cell cultures were screened by G418 selection agent (Corning, 30-234-CR, 750 µg ml^−1^) for 2 weeks. After screening, GFP^+^ cells were sorted into a 48-well plate automatically (BD, FACSAria III) so that each well contained only one cell. Sorted cells were cultured with 750 µg ml^−1^ of G418 for further selection. Clones that managed to survive and expand were transferred to another dish for further expansion, and a small aliquot was used to check GFP signal and cell morphology.

For some dual-color experiments, U2OS cells expressing LAMP1-EGFP were incubated in 1× PBS with LysoTracker DND99 (Invitrogen, L7528, 50 nM) for 5 minutes at 37 °C. Before imaging, cells were rinsed three times with 1× PBS.

### Reporting summary

Further information on research design is available in the Nature Portfolio Reporting Summary linked to this article.

### Disclaimer

The NIH and its staff do not recommend or endorse any company, product or service.

## Online content

Any methods, additional references, Nature Portfolio reporting summaries, source data, extended data, supplementary information, acknowledgements, peer review information; details of author contributions and competing interests; and statements of data and code availability are available at 10.1038/s41587-022-01651-1.

## Supplementary information

Supplementary Information Supplementary Figs. 1–20, Tables 1–5 and legends for Supplementary Videos 1–16

Reporting Summary

Supplementary Video 1 Images of autofluorescence from dirty coverslip during z stack with lateral illumination modulation (left, before mirror alignment) versus mostly axial modulation (right, after mirror alignment). See also Extended Data Fig. 2c.

Supplementary Video 2 3D projections of live vegetative *B. subtilis* stained with CellBrite Fix 488, marking membranes. Wide-field (top), 3D SIM (middle) and four-beam SIM reconstructions (bottom) are compared. See also Fig. 2b.

Supplementary Video 3 3D projections of fixed U2OS cell labeled with Tomm20 primary and rabbit Alexa Fluor 488 secondary antibodies, marking outer mitochondrial membranes. Wide-field (left), 3D SIM (middle) and four-beam SIM (right) reconstructions are compared. See also Fig. 2f,g.

Supplementary Video 4 Live U2OS cell stained with MitoTracker Green FM. First movie segment shows lateral views as a function of z (that is, ‘z stack’). Second movie segment shows the axial view as a function of lateral coordinate. See also Fig. 2h,i.

Supplementary Video 5 Four-beam SIM imaging of live, vegetative *B. subtilis* with GFP-DivIVA marker, projection view. See also Supplementary Fig. 11b.

Supplementary Video 6 Four-beam SIM imaging of fixed mouse LSECs with CellBrite Fix 488 label, marking membrane (cyan) and Alexa Fluor 568 phalloidin, marking actin filaments (magenta). See also Fig. 3h.

Supplementary Video 7 Time-lapse imaging of live U2OS cells stained with 200 nM Potomac Gold in four-beam SIM. Yellow arrow and rectangle highlight phototoxicity in one region. Red arrows highlight morphological changes of mitochondria between the 1st timepoint and the 10th timepoint. See also Supplementary Fig. 11g,h.

Supplementary Video 8 Time-lapse imaging of live U2OS cells expressing Tomm20-GFP marker. First movie segment shows 3D projections of mitochondrial dynamics after two-step denoising and isotropic prediction. Second movie segment shows higher magnification views of yellow rectangular region in the first movie segment. Raw low SNR 3D SIM reconstructions (top left), 1st step denoising results (top right), 2nd step denoising results (bottom left) and 3rd step isotropization (bottom right) are compared. Third movie segment shows axial planes indicated by yellow lines in the second movie segment. See also Fig. 5b–d.

Supplementary Video 9 Time-lapse imaging of live U2OS cells expressing LAMP1-EGFP marker. First movie segment shows 3D projections of lysosomal dynamics after two-step denoising and isotropic prediction. Second movie segment shows higher magnification views of yellow rectangular region in the first movie segment. Raw low SNR 3D SIM reconstructions (top left), 1st step denoising results (top right), 2nd step denoising results (bottom left) and 3rd step isotropization (bottom right) are compared. Third movie segment shows axial planes indicated by yellow lines in the second movie segment.

Supplementary Video 10 Time-lapse imaging of live U2OS cells expressing lysosomal marker LAMP1-GFP (cyan) and additionally labeled with LysoTracker Red to mark the lysosome interior (magenta). 3D projections of raw low SNR 3D SIM reconstructions (left) and two-step denoising and isotropization predictions (right) are compared. See also Fig. 5e.

Supplementary Video 11 Time-lapse imaging of additional live U2OS cell #2 expressing lysosomal marker LAMP1-GFP (cyan) and additionally labeled with LysoTracker Red to mark the lysosome interior (magenta). 3D projections of raw low SNR 3D SIM reconstructions (left) and two-step denoising and isotropization predictions (right) are compared.

Supplementary Video 12 Higher magnification view of maximum intensity projections of live U2OS cells in Supplementary Video 10. See also Fig. 5k.

Supplementary Video 13 Time-lapse imaging of live Jurkat T cells expressing EMTB 3× GFP after two-step denoising and isotropization prediction, perspective view. Two microtubule filaments are segmented (red and green), emphasizing that they are initially separated (yellow circles), merge together over the nucleus (yellow arrow) and separate again (yellow circles). See also Fig. 6e.

Supplementary Video 14 Time-lapse imaging of live Jurkat T cells expressing EMTB 3× GFP after two-step denoising and isotropization prediction. Microtubule organizing center is identified (cyan sphere, trajectory temporally coded as shown in color bar), and one microtubule filament is segmented (red), emphasizing their correlated, inward movement). Top: lateral perspective view. Bottom: axial *y*–*z* view. See also Fig. 6f.

Supplementary Video 15 Time-lapse imaging of live Jurkat T cells expressing EMTB 3× GFP after two-step denoising and isotropization prediction, emphasizing buckling of two microtubule filaments (red and yellow spheres). Top: lateral perspective view. Bottom: axial *y*–*z* view. See also Fig. 6g.

Supplementary Video 16 Time-lapse imaging of another live Jurkat T cells expressing EMTB 3× GFP after two-step denoising and isotropization prediction. One microtubule filament is segmented (red), and yellow arrows indicate buckling sites after sliding and contacting another filament. Left: lateral view. Right: zoomed-in perspective view. See also Supplementary Fig. 20.

Supplementary Data 1 Statistical source data for Supplementary Figs. 8–10 and 13 and Tables 1–4.

## Data Availability

The data that support the findings of this study are included in Extended Data Figs. 1–4, Supplementary Figs. 1–20 and Supplementary Videos 1–16. Some representative raw images from the figures (Figs. 1d, 2a,e,h, 3a–c,e,h, 4b,e, 5b,e and 6) and 3D SIM and four-beam SIM raw datasets (bacterial membranes and Tomm20 staining) are publicly available at https://zenodo.org/record/6727773. Other datasets (training data and intermediate data for deep learning) are available from the corresponding author upon reasonable request due to their large file size. Source data are provided with this paper.

## Source data

Source Data Fig. 1 Statistical Source Data for intensity profiles (**f**) and resolution (**g**)
Source Data Fig. 2 Statistical Source Data for intensity profiles (**d**)
Source Data Fig. 4 Statistical Source Data for intensity profiles (**d**)
Source Data Extended Data Fig. 3 Statistical Source Data for intensity profiles (**c**) and resolution (**d**)
Source Data Extended Data Fig. 4 Statistical Source Data for intensity profiles (**c** and **g**)

## Code availability

The microscope acquisition software used in this study was written in Python 3.7.1 and is available upon reasonable request. Code for the simulations of 2D and 3D OTF support, derivation of OTF from PSF, finding algorithm of SLM pattern and raw 3D SIM and four-beam SIM data reconstruction are available at https://github.com/eexuesong/SIMreconProject. CARE, RCAN and DenseDeconNet software were installed from https://github.com/CSBDeep/CSBDeep, https://github.com/AiviaCommunity/3D-RCAN and https://github.com/eguomin/regDeconProject/tree/master/DeepLearning.
