# Supplementary material for: Three-dimensional structured illumination microscopy with enhanced axial resolution
Source: Nat Biotechnol. 2023 Jan 26;41(9):1307–19. doi: 10.1038/s41587-022-01651-1 (PMC10497409; doi:10.1038/s41587-022-01651-1)
Supplement: Supplementary file 2 — Reporting Summary [file 41587_2022_1651_MOESM2_ESM.pdf]

nature portfolio

Corresponding author(s): Xuesong Li
Yicong Wu

Last updated by author(s): Dec 5, 2022

Reporting Summary

Nature Portfolio wishes to improve the reproducibility of the work that we publish. This form provides structure for consistency and transparency in reporting. For further information on Nature Portfolio policies, see our Editorial Policies and the Editorial Policy Checklist.

Statistics

For all statistical analyses, confirm that the following items are present in the figure legend, table legend, main text, or Methods section.

n/a	Confirmed	
☐	☒	The exact sample size (*n*) for each experimental group/condition, given as a discrete number and unit of measurement
☐	☒	A statement on whether measurements were taken from distinct samples or whether the same sample was measured repeatedly
☒	☐	The statistical test(s) used AND whether they are one- or two-sided<br>*Only common tests should be described solely by name; describe more complex techniques in the Methods section.*
☒	☐	A description of all covariates tested
☒	☐	A description of any assumptions or corrections, such as tests of normality and adjustment for multiple comparisons
☐	☒	A full description of the statistical parameters including central tendency (e.g. means) or other basic estimates (e.g. regression coefficient) AND variation (e.g. standard deviation) or associated estimates of uncertainty (e.g. confidence intervals)
☒	☐	For null hypothesis testing, the test statistic (e.g. *F*, *t*, *r*) with confidence intervals, effect sizes, degrees of freedom and *P* value noted<br>*Give P values as exact values whenever suitable.*
☒	☐	For Bayesian analysis, information on the choice of priors and Markov chain Monte Carlo settings
☒	☐	For hierarchical and complex designs, identification of the appropriate level for tests and full reporting of outcomes
☒	☐	Estimates of effect sizes (e.g. Cohen's *d*, Pearson's *r*), indicating how they were calculated

Our web collection on statistics for biologists contains articles on many of the points above.

Software and code

Policy information about availability of computer code

Data collection	The simulated OTF support images (Extended Data Fig. 1 and SI Fig. 1) were generated with MATLAB 2021b. The code is publicly available at (https://github.com/eexuesong/SIMreconProject/tree/main/OTF_simulation). The synthetic images (SI Fig. 13a) were generated with MATLAB 2019b. The code is publicly available at (https://github.com/MeatyPlus/Richardson-Lucy-Net/tree/main/Phantom_generate). Experimental images were collected with the home-built super-resolution structured illumination microscope (SIM) described in the Methods section of our paper. Microscope acquisition software was written in Python 3.7.1 and is available upon request.
Data analysis	CARE, RCAN and DenseDeconNet software was installed from GitHub (https://github.com/CSBDeep/CSBDeep; https://github.com/AiviaCommunity/3D-RCAN; https://github.com/eguomin/regDeconProject/tree/master/DeepLearning). No version numbers are defined for CARE, RCAN and DenseDeconNet. We used Python version 3.7.1 for all neural networks, and Tensorflow framework version 2.4.1, 1.13.1, 1.14.0 for CARE, RCAN and DenseDeconNet, respectively. Wiener reconstruction of SIM images was processed with MATLAB 2021b. The code is publicly available at (https://github.com/eexuesong/SIMreconProject/tree/main/Sirecon). Image analysis (e.g., calculation of SSIM, PSNR) code used in this study was written in MATLAB 2019a, and is available upon request. Aivia 10.2 and Imaris 9.8.2 were used to segment and track microtubule filaments in live Jurkat T cells.

For manuscripts utilizing custom algorithms or software that are central to the research but not yet described in published literature, software must be made available to editors and reviewers. We strongly encourage code deposition in a community repository (e.g. GitHub). See the Nature Portfolio guidelines for submitting code & software for further information.

Data

Policy information about availability of data

All manuscripts must include a data availability statement. This statement should provide the following information, where applicable:

- Accession codes, unique identifiers, or web links for publicly available datasets
- A description of any restrictions on data availability
- For clinical datasets or third party data, please ensure that the statement adheres to our policy

The data that support the findings of this study are included in Extended Data Figs. 1–4, Supplementary Figs. 1–20 and Supplementary Videos 1–16. Some representative raw images from the figures (Figs. 1d, 2a, 2e, 2h, 3a, 3c, 3h, 3b, 3e, 4b, 4e, 5b, 5e, 6) and 3D and 4-beam SIM raw datasets (bacterial membranes and Tomm20 staining) are publicly available at https://zenodo.org/record/6727773. Other datasets (training data and intermediate data for deep learning) are available from the corresponding author upon reasonable request due to their large file size. Source data are provided with this paper.

Human research participants

Policy information about studies involving human research participants and Sex and Gender in Research.

Reporting on sex and gender	N/A
Population characteristics	N/A
Recruitment	N/A
Ethics oversight	N/A

Note that full information on the approval of the study protocol must also be provided in the manuscript.

Field-specific reporting

Please select the one below that is the best fit for your research. If you are not sure, read the appropriate sections before making your selection.

☒ Life sciences ☐ Behavioural & social sciences ☐ Ecological, evolutionary & environmental sciences

For a reference copy of the document with all sections, see nature.com/documents/nr-reporting-summary-flat.pdf

Life sciences study design

All studies must disclose on these points even when the disclosure is negative.

Sample size

For resolution characterization, statistical results (mean +/- standard deviation) of FWHMs were obtained from N = 102, 100, 99 beads acquired with a 1.35 NA silicone oil immersion objective, and N = 85, 81, 78 beads acquired with a 1.27 NA water immersion objective, which are sufficient sample sizes for microscope resolution analysis (Fig. 1g, Extended Data Fig. 3d, SI Table 1) [1, 2]. Statistical results (mean +/- standard deviation) of spatial resolution comparisons using modified decorrelation analysis of 3D SIM, 4-beam SIM and DL isotropization were obtained from N = 50-100 lateral and axial images (SI Table 2), which are typical sample sized for imaging analysis [1, 2]. Statistical results (mean +/- standard deviation) of SSIM and PSNR were obtained from N = 11 lateral slices to compare the SSIM/PSNR between ground truth (GT) and predictions from different denoising methods (SI Fig. 18, SI Table 3). Statistical results (mean +/- standard deviation) of SSIM and PSNR were obtained from N = 14 lateral slices to compare the SSIM/PSNR between GT and predictions from two-step RCAN with and without an intermediate Wiener filter (SI Fig. 19, SI Table 4). With this sample size, the denoising improvement of two-step RCAN performance over other networks is obvious. For training the neural networks, the multi-step deep learning pipeline required a collection of ~50 volumetric pairs per network, and validated that the sample size is appropriate as the trained neural network models are not overfitting or underfitting [3].

[1] Tomer, Raju, et al. "Quantitative high-speed imaging of entire developing embryos with simultaneous multiview light-sheet microscopy." Nature methods 9.7 (2012): 755-763.
[2] Wu, Yicong, et al. "Multiview confocal super-resolution microscopy." Nature 600.7888 (2021): 279-284.
[3] https://docs.aws.amazon.com/machine-learning/latest/dg/model-fit-underfitting-vs-overfitting.html

Data exclusions

No data were excluded from the analysis.

Replication

The reproducibility of the experimental findings was verified by imaging distinct fixed and live samples with sample size N=10-100. Time-lapse imaging experiments were repeated at least 4 times, with similar results obtained each time. All attempts at replication were successful.

Randomization

In this study, samples were not allocated into different experimental groups.

Blinding

The investigators were not blinded to group allocation during data collection and data analysis. We don't think blinding is relevant in this study and we demonstrated the technique on distinct samples by collaborating with different research groups inside and outside NIH.

Reporting for specific materials, systems and methods

We require information from authors about some types of materials, experimental systems and methods used in many studies. Here, indicate whether each material, system or method listed is relevant to your study. If you are not sure if a list item applies to your research, read the appropriate section before selecting a response.

Materials & experimental systems

n/a	Involved in the study
☐	☒ Antibodies
☐	☒ Eukaryotic cell lines
☒	☐ Palaeontology and archaeology
☒	☐ Animals and other organisms
☒	☐ Clinical data
☒	☐ Dual use research of concern

Methods

n/a	Involved in the study
☒	☐ ChIP-seq
☒	☐ Flow cytometry
☒	☐ MRI-based neuroimaging

Antibodies

Antibodies used

(1) Microtubules (Figs. 3c-g, 4b-c, SI Figs. 11c-d, 13c, 19c) were labeled by mouse-α-alpha tubulin (Invitrogen, 32-2500, 1:100, B-5-1-2) and α-mouse Alexa Fluor 488 (Jackson Immuno Research, 715-547-003, 1:200, polyclonal).
(2) Mitochondria (Fig. 2e-g, SI Figs. 13d, Extended Data Fig. 4d-f, 17b-c, 18c) were labeled by rabbit-α-Tomm20 (Abcam, ab186735, 1:100, EPR15581-54) and α-Rabbit Alexa Fluor 488 (Jackson Immuno Research, 711-547-003, 1:200, polyclonal).
(3) T cell (Fig. 6, SI Fig. 20) activating antibody coating was performed by incubating slides in a 10 μg/mL solution of α-CD3 antibody (Thermo Fisher Scientific, 16-0039-85, Hit-3a, 1:100 dilution in 1X PBS).
(4) Vimentin (Fig. 3c-g) was labeled using rabbit-α-vimentin (Abcam, ab92547, 1:100, EPR3776) and α-Rabbit Alexa Fluor 594 (Jackson Immuno Research, 711-587-003, 1:200, polyclonal).
(5) Caveolae (Fig. 4e-k) were labeled with rabbit-α-PTRF (Abcam, 76919, 1:100, polyclonal) and α-Rabbit Alexa Fluor 568 (Invitrogen, A11011, polyclonal).
(6) Caveolin-1 EGFP (Fig. 4e-k, SI Fig. 15) was labeled using GFP-booster (ChromoTek, gb2AF488-50, 1:500, monoclonal, raised in alpaca, LOT 90917037AF1-02).

Validation

(1) Mouse-α-alpha tubulin (Invitrogen, 32-2500, 1:100, B-5-1-2)
(a) Vendor website: https://www.thermofisher.com/antibody/product/alpha-Tubulin-Antibody-clone-B-5-1-2-Monoclonal/32-2500
(b) Reference: A. York. Resolution Doubling in Live, Multicellular Organisms via Multifocal Structured Illumination Microscopy. Nat. Method (2013)

(2) Rabbit-α-Tomm20 (Abcam, ab186735, EPR15581-54)
(a) Vendor website: https://www.abcam.com/tomm20-antibody-epr15581-54-mitochondrial-marker-ab186735.html
(b) Vendor statement: Recombinant fragment. This information is proprietary to Abcam and/or its suppliers. Positive control: WB: HepG2, HeLa, SH-SY5Y, PC-12 and NIH 3T3 cell lysates; IHC-P: Human ovarian carcinoma and mouse cardiac muscle tissues; ICC/IF: HeLa cells; Flow Cyt (intra): HeLa cells; IHC-Fr: Mouse cardiac and small intestine tissues.
(c) Reference: Kuang W. SLC22A14 is a mitochondrial riboflavin transporter required for sperm oxidative phosphorylation and male fertility. Cell Rep (2021)

(3) α-mouse Alexa Fluor 488 (Jackson Immuno Research, 715-547-003, polyclonal)
(a) Vendor website: https://www.jacksonimmuno.com/catalog/products/715-547-003
(b) Reference: C. Liu. The Role of Bone Morphogenetic Protein 4 in Microglial Polarization in the Process of Neuropathic Pain. Journal of Inflammation Research (2022)

(4) α-Rabbit Alexa Fluor 488 (Jackson Immuno Research, 711-547-003, polyclonal)
(a) Vendor website: https://www.jacksonimmuno.com/catalog/products/711-547-003
(b) Reference: X. Han. A polymer index-matched to water enables diverse applications in fluorescence microscopy. Lab on a chip (2021)

(5) α-CD3 (Thermo Fisher Scientific, 16-0039-85, Hit-3a)
(a) Vendor website: https://www.thermofisher.com/antibody/product/CD3-Antibody-clone-HIT3a-Monoclonal/16-0039-85
(b) Vendor statement: The HIT3a monoclonal antibody reacts with human CD3e, a 20 kDa subunit of the TCR complex. Along with the other CD3 subunits gamma and delta, the epsilon chain is required for proper assembly, trafficking and surface expression of the TCR complex. CD3 is expressed by thymocytes in a developmentally regulated manner and by all mature T cells. Crosslinking of TCR with HIT3a initiates an intracellular biochemical pathway resulting in cellular activation and proliferation.
(c) Reference: B. Senechal. Expansion of regulatory T cells in patients with Langerhans cell histiocytosis. Plos Medecine (2007)

(6) rabbit-α-vimentin (Abcam, ab92547, EPR3776)
(a) Vendor website: https://www.abcam.com/vimentin-antibody-epr3776-cytoskeleton-marker-ab92547.html
(b) Vendor statement: Synthetic peptide. This information is proprietary to Abcam and/or its suppliers.
(c) Reference: Y. Li Single-cell transcriptome profiling of the vaginal wall in women with severe anterior vaginal prolapse. Nat Commun (2021)

(7) α-Rabbit Alexa Fluor 594 (Jackson Immuno Research, 711-587-003, 1:200, polyclonal)

(a) Vendor website: https://www.jacksonimmuno.com/catalog/products/711-587-003
(b) Reference: D. Bradshaw. Genetic inactivation of SARM1 axon degeneration pathway improves outcome trajectory after experimental traumatic brain injury based on pathological, radiological, and functional measures. Acta Neuropathologica Communications

(8) rabbit-α-PTRF (Abcam, 76919, 1:100, polyclonal)
(a) Vendor website: https://www.abcam.com/ptrf-antibody-ab76919.html
(b) Vendor statement: Synthetic peptide corresponding to a region between residue 125 and 175 of human PTRF (NP_036364.2)
(c) Reference: F. Wu. Development and Verification of a Hypoxic Gene Signature for Predicting Prognosis, Immune Microenvironment, and Chemosensitivity for Osteosarcoma. Front Mol Biosci (2021)

(9) α-Rabbit Alexa Fluor 568 (Invitrogen, A11011, polyclonal)
(a) Vendor website: https://www.thermofisher.com/antibody/product/Goat-anti-Rabbit-IgG-H-L-Cross-Adsorbed-Secondary-Antibody-Polyclonal/A-11011
(b) Reference: I. Ribeiro. Spatial and temporal control of expression with light-gated LOV-LexA. G3 (2022)

(10) GFP-booster (ChromoTek, gb2AF488-50, 1:500)
(a) Vendor website: https://www.ptglab.com/products/GFP-Booster-Alexa-Fluor-488-gb2AF488.htm
(b) Reference: B. Hubner. Correlative microscopy of individual cells: sequential application of microscopic systems with increasing resolution to study the nuclear landscape. Methods Mol Biol (2013)

Eukaryotic cell lines

Policy information about cell lines and Sex and Gender in Research

Cell line source(s)	C57BL/6 mouse primary liver sinusoidal endothelial cells (LSECs, commercial products purchased from Cell Biologics, cat: C57-6017), C57BL/6N mouse embryonic fibroblasts (MEFs, gift from Oliver Daumke's lab), human T lymphocyte (Jurkat E6-1, ATCC TIB-152, gift from Dr Lawrence E. Samelson, NIH), and human osteosarcoma (U2OS, ATCC HTB-96) cell lines were used in this study.
Authentication	None of the cell lines used were authenticated.
Mycoplasma contamination	The cell lines were not tested for mycoplasma contamination.
Commonly misidentified lines (See ICLAC register)	No commonly misidentified cell lines were used in the study.
